# ﻿Resolving issues in the genus *Dioxys* (Hymenoptera, Megachilidae, Dioxyini) in the West Palaearctic with a new identification key

**DOI:** 10.3897/zookeys.1226.138377

**Published:** 2025-02-10

**Authors:** T. J. Wood

**Affiliations:** 1 Naturalis Biodiversity Center, Darwinweg 2, 2333 CR, Leiden, Netherlands Naturalis Biodiversity Center Leiden Netherlands

**Keywords:** Genital capsule, new species, North Africa, parasitic bees, revisionary taxonomy

## Abstract

The bee genus *Dioxys* is widely distributed across the Holarctic from the Mediterranean basin to western North America but is species-poor, and individual species can prove challenging to identify. Consequently, there has been a lack of consensus as to how many species actually exist. In the West Palaearctic, the number of species has varied from six to ten, depending on the worker. Due to a previously incorrect assessment of publication dates, *Dioxysrotundatus* Pérez, 1884, **sp. resurr.** is restored as the senior synonym of *Dioxysmoestus* Costa, 1884, **syn. nov.** The relationship between this species and *Dioxysatlanticus* Saunders, 1904 is clarified, with the latter restricted to the islands of Gran Canaria and Tenerife (Spain). *Dioxysrufipes* Morawitz, 1875 is considered part of the West Palaearctic fauna, replacing “*D.moestus*” sensu Warncke (1977) in the eastern Mediterranean. *Dioxysmontanus* Heinrich, 1977, **sp. resurr.** is revalidated from synonymy with *Dioxyscinctus* (Jurine, 1807). *Dioxyspumilus* Gerstäcker, 1869 is found to consist of four species, *D.pumilus* (eastern Mediterranean), *Dioxysvaripes* De Stefani, 1887, **sp. resurr.** (western Mediterranean), *Dioxyscypriacus* Popov, 1944, **sp. resurr.** (Cyprus), and *Dioxyshermonensis***sp. nov.** (Israel: Mount Hermon). A neotype is designated for *D.varipes*, and *Dioxysfalsificus* Engel, 2023, **syn. nov.** is synonymised with it. This contribution produces a total of 13 species for the West Palaearctic region, and illustrates the degree to which persistent taxonomic problems exist even within small bee genera.

## ﻿Introduction

*Dioxys* Lepeletier & Serville, 1825 is the largest genus in the species-poor Megachilid tribe Dioxyini Cockerell, 1902. The placement of this tribe, and even its recognition as a tribe (or not) has varied over time (e.g., Popov 1947; Roig-Alsina and Michener 1993; Gogala 1995; Michener 2007; Litman et al. 2011; 2013; Gonzalez et al. 2012). Based on molecular evidence, Dioxyini is best placed at the base of subfamily Megachilinae (Litman et al. 2011; 2013). However, regardless of its overall placement, authors have been consistent as recognising the grouping as monophyletic due to the group’s distinctive morphology (Gonzalez et al. 2012), namely the presence of a median tubercle on the metanotum (although this is absent in the genus *Eudioxys* Mavromoustakis, 1963 and greatly reduced in *Metadioxys* Popov, 1947) and its extremely reduced sting.

Within the Dioxyini, the genus-level concepts have also varied significantly. As the oldest name available, this means that concepts of the genus *Dioxys* have varied over time. Other than the five species found in North America (Hurd 1958) which are currently placed in *Dioxys* sensu stricto, all other lineages within Dioxyini are restricted to the Old World (Michener 2007). Whilst some workers have treated *Dioxys* as a broad genus and have used available groupings as subgenera (e.g., Warncke 1977), the currently accepted approach is to consider multiple genera (Michener 1996; Michener 2007; Engel, 2023). Currently, nine genera are used within Dioxyini for a total of approximately 33 species (due to species-level uncertainties within the genus *Dioxys*). These genera are *Allodioxys* Popov, 1947 (four species), *Aglaoapis* Cameron, 1901 (two species), *Dioxys* (~15 species), *Ensliniana* Alfken, 1938 (one species), *Eudioxys* (two species), *Metadioxys* (three species), *Notodioxys* Engel, 2023 (one species), *Paradioxys* Mocsáry, 1894 (two species), and *Prodioxys* Friese, 1914 (three species) (Popov, 1947; Michener, 2007; Engel, 2023). Whilst it may seem slightly excessive to have nine genera for such a small number of species, that discussion is beyond the scope of the current study (although see comments in Wood 2023).

This current study is twice limited: it focuses only on the species currently placed in the genus *Dioxys* sensu stricto (following Michener 2007), and it focuses only on the West Palaearctic region, as classically defined. This excludes Central Asia, and hence the three *Dioxys* species known only from Central Asia, specifically Turkmenistan and Uzbekistan (*Dioxysdistinguendus* Popov, 1936; *Dioxysmodestus* Popov, 1936; *Dioxysturkestanicus* Popov, 1936) are not treated here, since they are known from only their type series and no further publications have been made on them.

Within the West Palaearctic region, the major revisionary work is Warncke (1977), following Popov (1936, 1944, 1947) who described new species from the West and Central Palaearctic. As is typical of Warncke’s work, he placed all Dioxyini into a single genus, adopted broad species concepts, placed certain taxa in combination with others as subspecies, and did not precisely cite specimen details or institutional repositories. However, he did propose several synonymies which have more or less gained acceptance, and produced relatively robust species concepts with an identification key which broadly works, as long as one has access to a good reference collection. Subsequently, this work has been built on (Tkalců 2001; Bogusch 2023), but some of the original issues that were never resolved by the work of Warncke remain. Moreover, some taxonomic decisions made by recent works (Bogusch 2023; Engel 2023) have not full resolved some of the problems inherent in Warncke’s work or have created new names which must be integrated into a broader West Palaearctic framework. There is therefore a need for a deeper analysis of the genus *Dioxys* in the West Palaearctic in order to resolve these issues before they become embedded in the literature, and to produce a newly revised comprehensive identification key so that consistent species concepts can be employed in this genus.

## ﻿Materials and methods

First, it is necessary to comment on the spelling of name placed in combination with the genus *Dioxys*. The genus has a masculine gender, as the suffix -*oxys* is the masculine singular nominative form of a Greek adjective, and hence genera ending in “-*oxys*” are unambiguously masculine, regardless of their author’s intent (Doug Yanega quoted in Nieuwenhuijsen 2020). Therefore, masculine terminations are used for each *Dioxys* name herein. Original spellings are used only in the chresonymy for each species.

Species were examined morphologically; no genetic data were generated for this work. The work of Wood (2023) and the genetic data generated therein are briefly referenced. The species concepts presented are therefore based primarily on morphology. Morphological terminology follows Michener (2007). All specimens were identified by myself, unless explicitly stated; specimens identified by others were visually inspected and validated. The following abbreviations are used in the species descriptions: **A** = antennal segments, **S** = metasomal sterna, and **T** = metasomal terga.

Specimens were measured from the centre of the clypeus at the front of the head to the apical tip of the metasoma and rounded to the nearest 0.5 mm with a ruler. Photographs were taken using an Olympus E-M1 Mark II with a 60 mm macro lens. Additional close-ups were taken with the addition of a Mitutoyo M Plan Apo 10X infinity corrected objective lens in combination with an Olympus M.Zuiko 2x teleconverter lens, a 10 mm Kenko DG extension tube, and a Meike MK-P-AF3B 10 mm extension tube. Photographs were stacked using Helicon Focus B (HeliconSoft, Ukraine) and plates were prepared in GNU Image Manipulation Program (GIMP) 2.10. Post-processing of some images was made in Photoshop Elements (Adobe Systems, USA) to improve lighting to highlight specific characters.

Due to the large number of taxonomic changes, and the complexity of specimen identification in this genus, a revised identification key loosely based on Warncke (1977) is presented at the beginning of the results. Following this, species are presented alphabetically. For species distributions, countries marked with an asterisk indicate the first published record for that country. This is complicated due to the variable species concepts that have been used, and the lack of precise specimen information presented by Warncke (1977), making it difficult to locate and re-identify his examined specimens.

### ﻿Abbreviations used

**HNHM** Hungarian Natural History Museum, Budapest, Hungary

**IENU** Istituto di Entomologia, Università degli Studi, Naples, Italy

**LRC** Personal collection of Francisco La Roche, San Cristóbal de La Laguna, Tenerife, Spain

**MNHN** Muséum national d’Histoire naturelle, Paris, France

**MSCA** Personal collection of Maximillian Schwarz, Ansfelden, Austria

**MSVI** Personal collection of Marco Selis, Viterbo, Italy

**NHMUK** Natural History Museum, London, United Kingdom

**NMW** Naturhistorisches Museum Wien, Vienna, Austria

**OÖLM** Oberösterreiches Landesmuseum, Linz, Austria

**OUMNH** Oxford University Museum of Natural History, Oxford, United Kingdom

**RMNH** Naturalis Biodiversity Center, Leiden, the Netherlands

**SEMC** University of Kansas Natural History Museum, Lawrence, Kansas, USA

**TJWC** Personal collection of T.J. Wood, Leiden, the Netherlands

**USNM** Smithsonian National Museum of Natural History, Washington D.C., USA

**ZISP** Zoological Institute of the Russian Academy of Sciences, St. Petersburg, Russia

**ZMHB** Museum für Naturkunde, Berlin, Germany

**ZSM** Zoologische Staatssammlung München, Munich, Germany

## ﻿Results

A total of 414 *Dioxys* specimens from 23 West Palaearctic countries or territories were examined, comprising 232 females and 182 males of 13 species. Revision of the genus has revealed that several taxonomic changes are necessary for a variety of reasons, including incorrect publication dates and a lack of examination of the male genital capsule. Due to the complexity of specimen identification, it is considered most useful to present an identification key here at the beginning of the results section, so that readers can understand the morphological differences between the species treated here. The necessary taxonomic changes are formalised below.

The key is most clearly structurally based on the one presented by Warncke (1977), but since he considered a total of only six *Dioxys* sensu stricto species for the West Palaearctic region, substantial modifications have been made. Additional images and illustrations can be found in Popov (1936), Warncke (1977), and Bogusch (2023), though care should be taken to integrate the numerous taxonomic changes made both during the 20^th^ century and herein.

### ﻿Identification key for the genus *Dioxys* in the West Palaearctic region

The females of *D.lanzarotensis* Tkalců, 2001 and *D.hermonensis* sp. nov. are unknown. These species are therefore absent from the female part of the key.

**Table:** 

1	Females; metasoma with 6 tergal segments, antennae with 12 segments	**2**
–	Males; metasoma with 7 tergal segments, antennae with 13 segments	**11**
2	Specimens with bright red mesosomal pubescence, terga without apical hairbands (with weak hairs, but these not obviously contrasting the colouration of the metasoma)	**3**
–	Specimens with pale mesosoma pubescence, terga with clear pale apical hairbands which clearly contrast the underlying colouration of the metasoma (Figs 5E, 11C, 12D, 13E)	**4**
3	Mandible medially strongly thickened and bulging, giving the impression that the mandibles are medially bent. A4 quadrate, A5–A12 longer than broad. Scutum with long upstanding hairs. T1–T3 orange-red, T4–T6 black	***D.chalicodus* Lucas**
–	Mandible normal, not noticeably thickened or bent. A4 twice as wide as long, A5–A12 subquadrate. Scutum with short bristly hair. All tergal segments red	***D.ardens* Gerstäcker**
4	In dorsal view, outline of S6 more or less rectangular, clearly broader than long, and with the ventrolateral corners clearly visible on either side of T6 (Fig. 1A). T6 with a weak median emargination. Highly variable in size, between 5–12 mm in length. The most commonly encountered and widespread member of the genus	***D.cinctus* (Jurine)**
–	In dorsal view, S6 more or less rounded or weakly triangular, never rectangular, at most with parts of S6 slightly protruding beyond margin of T6 (e.g., in *D.montanus* Heinrich), never with rounded corners clearly visible on either side of T6 (Figs 1B–F, 5F, 7D, H, 8D, 11D, 13F). Apical margin of T6 entire. Not so variable in size, usually < 10 mm in length (*D.montanus* with body length of 10 mm)	**5**
5	Head and mesosoma covered with very short adpressed pubescence, giving the bee a greyish appearance (Fig. 2A–D). If in doubt, consider the surface of the top of the head adjacent to the ocellar triangle; here, the grey to pale brown hairs are clearly bent, lying down parallel to the integumental surface and strongly contrasting it (Fig. 2A)	**6**
–	Head and mesosoma not covered with very short adpressed pubescence, instead with upstanding and usually pale to whitish hairs. If in doubt, the surface of the top of the head adjacent to the ocellar triangle never has adpressed hairs, hairs here clearly upstanding (Fig. 2E, F)	**8**
6	T6 with surface finely punctate, without punctures becoming interlinked and also forming longitudinal grooves (Fig. 1C). Adpressed hairs of head and mesosoma relatively sparse, hairs individually relatively thin, not forming thick patches (Fig. 2A). Restricted to the eastern Mediterranean from Croatia eastwards	***D.rufipes* Morawitz**
–	T6 with surface densely punctate, punctures almost becoming confluent, forming longitudinal grooves (Fig. 1D–F). Adpressed hairs of head and mesosoma relatively dense, hairs individually thicker due to stronger plumosity, often forming thick patches (Fig. 2B–D). Found in both the eastern and western Mediterranean; specimens with relatively sparser pubescence can be found in the western Mediterranean, but these have a geographic range which does not coincide with *D.rufipes*, and they display a densely punctate T6 with longitudinal grooves	**7** ^1^
7	T6 often extensively marked with red; surface marginally comparatively narrow, width:length ratio of disc of T6 generally 1.5:1 (Fig. 1D). Found in the eastern Mediterranean from southern Greece eastwards (but not including Cyprus)	***D.pumilus* Gerstäcker**
–	T6 often extensively marked with red; surface marginally comparatively narrow, width:length ratio of disc of T6 generally 1.5:1 (Fig. 1E). Found on the island of Cyprus only	***D.cypriacus* Popov**
–	T6 usually dark, sometimes mixed slightly with red; slightly but comparatively broader, width:length ratio of disc of T6 generally 1.6:1 (Fig. 1F). Found in the western Mediterranean as far east as Sicily and Libya	***D.varipes* De Stefani**
8	Shape of S6 distinctive, weakly triangular with lateral margins slightly inwardly bowed, apex of S6 more or less truncate and clearly projecting beyond apical margin of T6 in dorsal view (Fig. 11D, contrasting lateral margins of S6 which remain extremely close to the outer margin of T6). Restricted to mountainous areas in south-western and central Turkey	***D.montanus* Heinrich**
–	Shape of S6 not noticeably different to outline of T6, both evenly rounded semi-circular (Figs 2B, 5F). Found in the western Mediterranean and North Africa only	**9**
9	A3 almost as long as A4+5; A6 distinctly subquadrate. Clypeus strongly domed and finely punctate, punctures half as dense as those on vertex. Larger, 8 mm in length. Found only in Morocco and Algeria	***D.heinrichi* Warncke**
–	A3 clearly shorter than A4+5; A6 slightly longer than broad. Clypeus only weakly domed, punctures of equal strength and density as those on vertex. Smaller, typically 5–7 mm in length. Found in Western Europe, North Africa, and the Canary Islands	**10**
10	T6 with punctures of variable size (smallest on the edge of the disc, becoming larger medially), but the punctures are clear and well-defined, with shiny interspaces (Figs 1B, 7D, 8D), giving a well-ordered impression, though can be slightly chaotic (Fig. 7H). Width:length ratio of disc of T6 1.8–1.9:1, less than twice as wide as long. Usually with the metasoma partially marked with red, but sometimes with the whole body integument black (e.g., some specimens from Sardinia). Found across the western Mediterranean and North Africa to Egypt but not the Canary Islands	***D.rotundatus* Pérez**
–	T6 with punctures of variable size (smallest on the edge of the disc, largest medially) but punctures irregular and shallow, very poorly defined (Fig. 5F). Punctures are placed slightly closer together which, when combined with their shallow nature, means that it is difficult to distinguish between the shiny interspaces and the punctures themselves, giving a chaotic overall impression. Width:length ratio of disc of T6 2.1:1, more than twice as wide as long. Integument of the body always entirely black (Fig. 5B–E). Currently known only from the Canary Islands (Tenerife and Gran Canaria)	***D.atlanticus* Saunders**
11(1)	Specimens with bright red mesosomal pubescence, terga without apical hairbands (with weak hairs, but these not obviously contrasting the colouration of the metasoma)	**12**
–	Specimens with pale mesosoma pubescence, terga with clear pale apical hairbands which clearly contrast the underlying colouration of the metasoma (Figs 6D, 9D, 10E, 11G, 14C)	**13**
12	Marginal area of S4 medially emarginate with lateral teeth, therefore appearing bidentate. Metasoma basally orange-red (typically T1, T2), apically black (typically T3–6)	***D.chalicodus* Lucas**
–	Marginal area of S4 straight, without any teeth. Metasoma uniformly red	***D.ardens* Gerstäcker**
13	Scutum with short hairs; viewed in profile, the length of these hairs shorter than or only slightly exceeding the diameter of a lateral ocellus (Figs 10D, 14B). This character can sometimes be a little variable (perhaps due to local climatic conditions, with shorter hairs shown by specimens from hotter and drier locales), and so if in doubt, then the species usually goes here; when the hairs are clearly longer than the diameter of a lateral ocellus, they are typically conspicuously longer. Hairs typically brownish (*pumilus*-group)	**14**
–	Scutum with longer hairs; viewed in profile, the length of these hairs clearly much longer than the diameter of a lateral ocellus (Figs 6B, 9B, 11F) . Hairs typically white to grey	**17**
14	Genital capsule with penis valves apically thickened, outer margin with rounded lateral projection, apexes of penis valves therefore not appearing triangular (Fig. 3C, E). Found in the western Mediterranean (east to Sicily and Libya) or Cyprus	**15**
–	Genital capsule with penis valves apically produced into essentially triangular shapes, with strong angle on outer margin (Fi. 3A, G). Found in the eastern Mediterranean (Greece eastwards to Turkey and the Levant)	**16**
15	Apex of penis valves produced into a relatively weak point (Fig. 3C). Apical margin of S4 variable, with a broad medial section slightly projecting anteriorly, this medial section with its apical margin straight to weakly emarginate (Fig. 3D). Found in the western Mediterranean (eastwards to Sicily and Libya)	***D.varipes* De Stefani**
–	Apex of penis valves produced into a relatively sharp point (Fig. 3E). Apical margin of S4 widely emarginate with a small obscure tooth placed medially (Fig. 3F). Found only on the island of Cyprus	***D.cypriacus* Popov**
16	Apical margin of S4 medially emarginate, with a distinct tooth medially (Fig. 3B). S5 with lateral margins rounded. Widespread in the eastern Mediterranean	***D.pumilus* Gerstäcker**
–	Apical margin of S4 straight (Fig. 3H). S5 with lateral margins produced into short blunt teeth (Fig. 10F). Currently found only on Mount Hermon (Israel)	***D.hermonensis* sp. nov.**
17	Genital capsule with penis valves more or less parallel-sided, apically tapering to sharp points (Fig. 4A). S3 with a long hairband, this 3 × longer than the diameter of a lateral ocellus and strongly contrasting the much shorter hairband on S2 (Fig. 4B). S4 apically with broad and rounded semi-circular projection, never emarginate (Fig. 4B). Found in the eastern Mediterranean	***D.rufipes* Morawitz**
–	Genital capsule otherwise, either with penis valves produced into clear triangular shapes apically (Fig. 4C, E), or with apexes broadened (Fig. 9F), but never with the penis valves parallel-sided and apically tapering into sharp points. S3 with apical hairband not so long (typically not ≥2 × the diameter of a lateral ocellus in length) or so strongly contrasting apical hairband of S2 (Fig. 4D, F). S4 apical margin variable, but never with semi-circular projection (either straight or emarginate medially, Figs 4D, F, 6F, 9E, 11H). Distribution variable	**18**
18	Apical margin of S4 straight, without a median emargination (Figs 9E, 11H)	**19**
–	Apical margin of S4 emarginate, either deeply or shallowly; emargination flanked by two projecting teeth of various lengths, teeth either clearly elongate or short and stubby (Figs 4D, F, 6F)	**20**
19	S4 with sparse and weak apical hair fringe, barely covering the underlying surface (Fig. 9E). S6 ventrally with very short hair fringe, these hairs shorter than the diameter of a lateral ocellus. Found in Morocco and Algeria	***D.heinrichi* Warncke**
–	S4 with dense and long apical hair fringe that obscures the underlying surface (Fig. 11H). S6 ventrally with a longer hair fringe, these hairs longer than the diameter of a lateral ocellus. Restricted to mountainous areas in south-western and central Turkey	***D.montanus* Heinrich**
20	S4 with a very wide and shallow emargination, occupying 1/3 of the width of the segment, this wide emargination flanked by strong projecting teeth (see illustrations in Bogusch 2023). Terga sparsely punctate, punctures separated by 1–3 puncture diameters. Restricted to the island of Lanzarote	***D.lanzarotensis* Tkalců**
–	S4 with emargination variable in strength, but always narrow, typically occupying 1/5 of the width of the segment; flanking teeth variable (Figs 4D, F, 6F). Terga more densely and regularly punctate, punctures separated by one puncture diameter. Distribution otherwise	**21**
21	S4 with shallow to weak median emargination, laterally flanked by short and barely projecting teeth (Fig. 4D)	***D.cinctus* (Jurine)**
–	S4 with a deep emargination flanked by long projecting teeth (Figs 4F, 6F)	**22**
22	T5 and T6 densely and regularly punctate, with punctures clearly visible. Found across the western Mediterranean and North Africa to Egypt but not the Canary Islands	***D.rotundatus* Pérez**
–	T5 and T6 shallowly punctate, punctures so shallow that they begin to disappear into the underlying surface, the surface of T6 therefore almost appearing smooth and polished (Fig. 6E). Currently known only from the Canary Islands (Tenerife and Gran Canaria)	***D.atlanticus* Saunders**

#### ﻿1. Dioxys ardens

Taxon classificationAnimaliaHymenopteraMegachilidae

Gerstäcker, 1869

D6069F34-D2DB-5D8B-A70D-7C4E42E7E5FE

Dioxys ardens Gerstäcker, 1869: 166, ♀ [Spain, ZMHB, not examined].
Dioxys rufispina Pérez, 1895: 26, ♀ [Algeria, MNHM, not examined].

##### Material examined.

**Israel** • 10♂, 3♀; Hasharon, Ma’agan Mikhael; 8–23 Mar. 1990; R. Leys leg.; RMNH; ZMA.INS.5103984–ZMA.INS.5103996; **Jordan** • 1♂, 2♀; 70 km NE Aqaba (Strasse nach Amman); 14 Apr. 1989; J. Gusenleitner leg.; M. Schwarz det.; OÖLM • 2♀; 80 km NE Aqaba (Strasse nach Amman); 11 Apr. 1989; J. Gusenleitner leg.; M. Schwarz det.; OÖLM; **Morocco** • 1♂; 20 km N Tiznit; 24 Mar. 1987; J. Gusenleitner leg.; K. Warncke det.; OÖLM • 1♂; Biougra, 30 km SE Agadir; 1 Apr. 1987; J. Gusenleitner leg.; M. Schwarz det.; OÖLM • 1♀; Foret Mamora, S de Kenitra; 17 Mar. 1961; Lindberg leg.; RMNH; RMNH.INS.1660517; **Spain** • 1♂; E Estepona; 1 Apr. 1985; H. Wolf leg.; RMNH; RMNH.INS.1660489 • 4♂, 2♀; E Estepona; 1 Apr. 1985; H. Wolf leg.; K. Warncke det.; OÖLM; **Tunisia** • 1♀; Is. Djerba, 14 km SE Houmt Souk; 27 Mar. 1992; J. Gusenleitner leg.; M. Schwarz det.; OÖLM.

**Figure 1.**
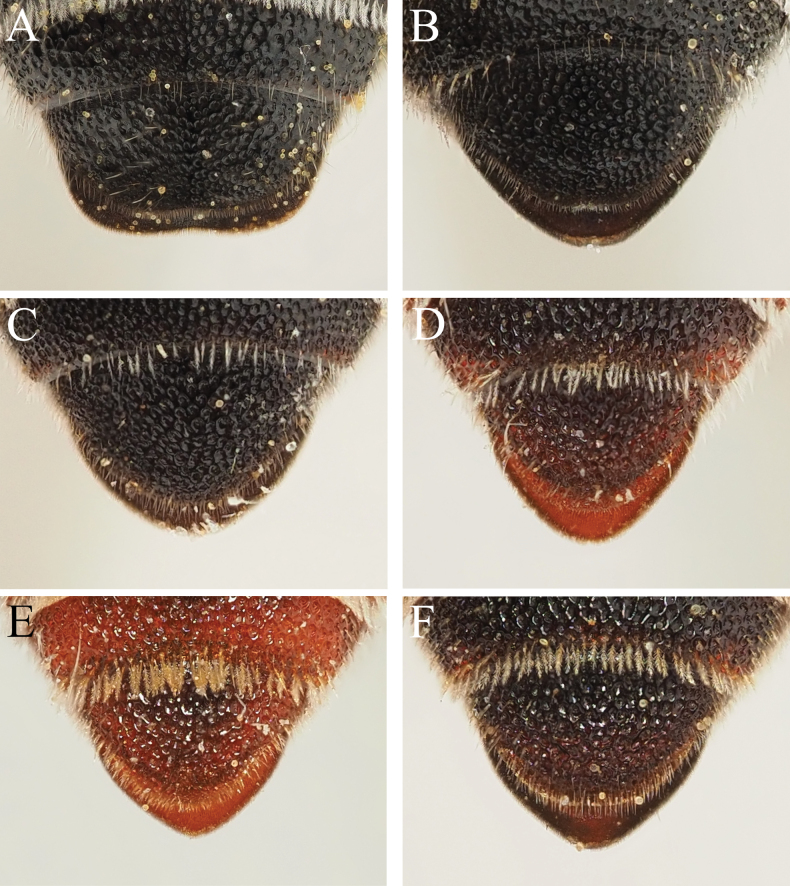
Female *Dioxys* species, T6, dorsal view **A***Dioxyscinctus* (Jurine, 1807) (Spain) **B***Dioxysrotundatus* Pérez, 1884 (Morocco) **C***Dioxysrufipes* Morawitz, 1875 (Turkey) **D***Dioxyspumilus* Gerstäcker, 1869 (Greece: Kos) **E***Dioxyscypriacus* Popov, 1944 (Cyprus) **F***Dioxysvaripes* De Stefani, 1887 (Morocco).

**Figure 2.**
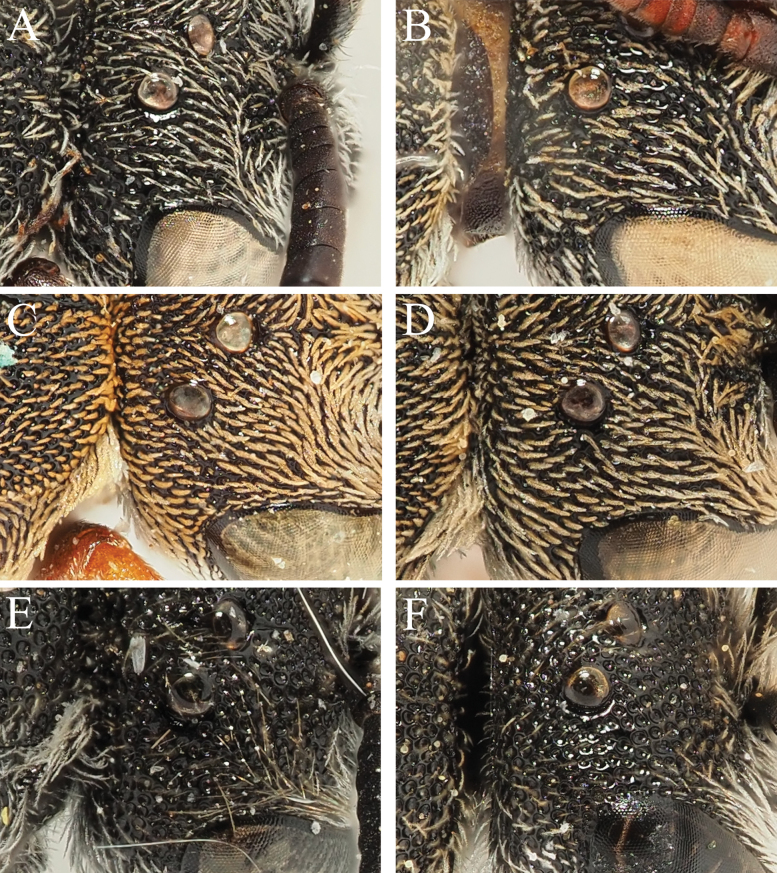
Female *Dioxys* species, space between ocellar triangle and compound eye, dorsal view **A***Dioxysrufipes* Morawitz, 1875 (Turkey) **B***Dioxyspumilus* Gerstäcker, 1869 (Greece: Kos) **C***Dioxyscypriacus* Popov, 1944 (Cyprus) **D***Dioxysvaripes* De Stefani, 1887 (Morocco) **E***Dioxysatlanticus* Saunders, 1904 (Spain: Gran Canaria) **F***Dioxysrotundatus* Pérez, 1884 (Morocco).

##### Distribution.

Portugal, Spain, Morocco, Algeria, Tunisia, ?Libya, Israel, Jordan* (Warncke 1977; Kuhlmann et al. 2014; Bogusch 2023).

##### Distributional notes.

The distribution of this species requires some clarification. Bogusch (2023) lists Portugal, Spain, and North Africa from Morocco to Libya, adding specifically that “This species is known only from several records from southern parts of Spain and one record from Portugal”, but without giving any specimen details. *Dioxysardens* has not previously been recorded from Portugal (Baldock et al. 2018); the specimen from Portugal is in the collection MSCA with the label information “Port. Alva” (P. Bogusch, pers. comm. December 2024), but this is difficult to interpret as this could refer to Vila Alva or Barca de Alva, or the Alva river. It can be tentatively accepted as present in Portugal, though further study is required to precisely establish its range. The occurrence of *D.ardens* in Libya is highly plausible given its overall distribution, but again precise specimen details would allow for greater confidence in its listing. Finally, Kuhlmann et al. (2014) list *D.ardens* from Israel without published specimen records, but the occurrence of this species in southern Israel can be confirmed through examined specimens, as well as its presence in neighbouring Jordan.

**Figure 3.**
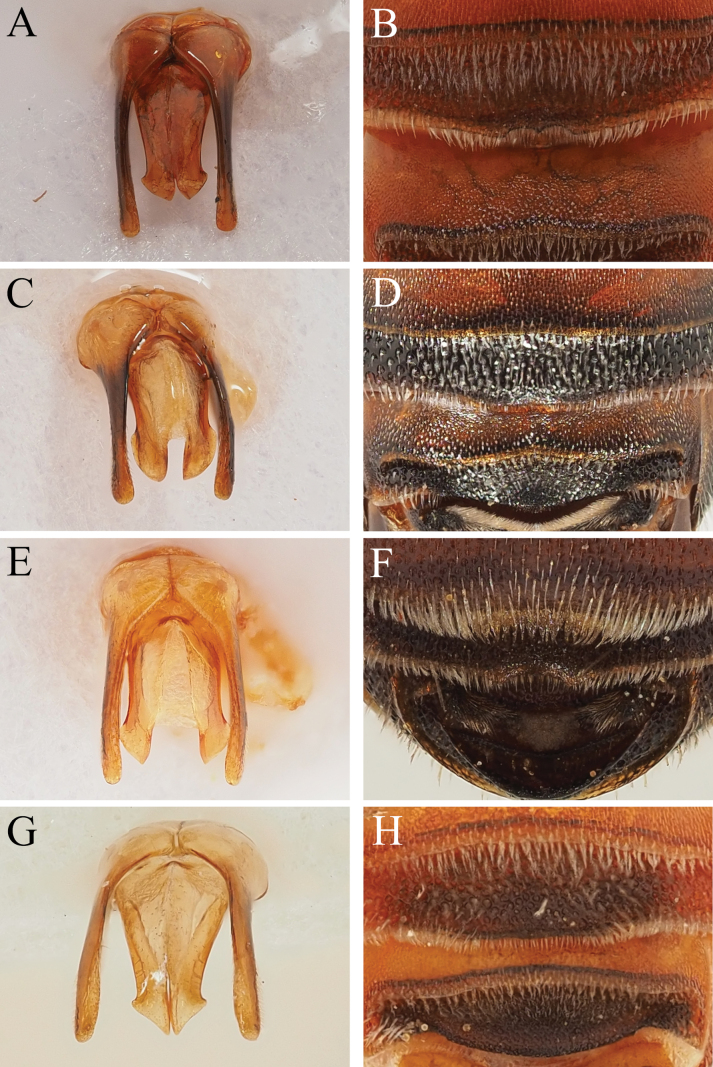
Male *Dioxys* in the *pumilus*-group, genital capsule and S4 **A, B***Dioxyspumilus* Gerstäcker, 1869 (Greece: Rhodes) **C, D***Dioxysvaripes* De Stefani, 1887 (Morocco) **E, F***Dioxyscypriacus* Popov, 1944 (Cyprus) **G, H***Dioxyshermonensis* sp. nov. (Israel).

**Figure 4.**
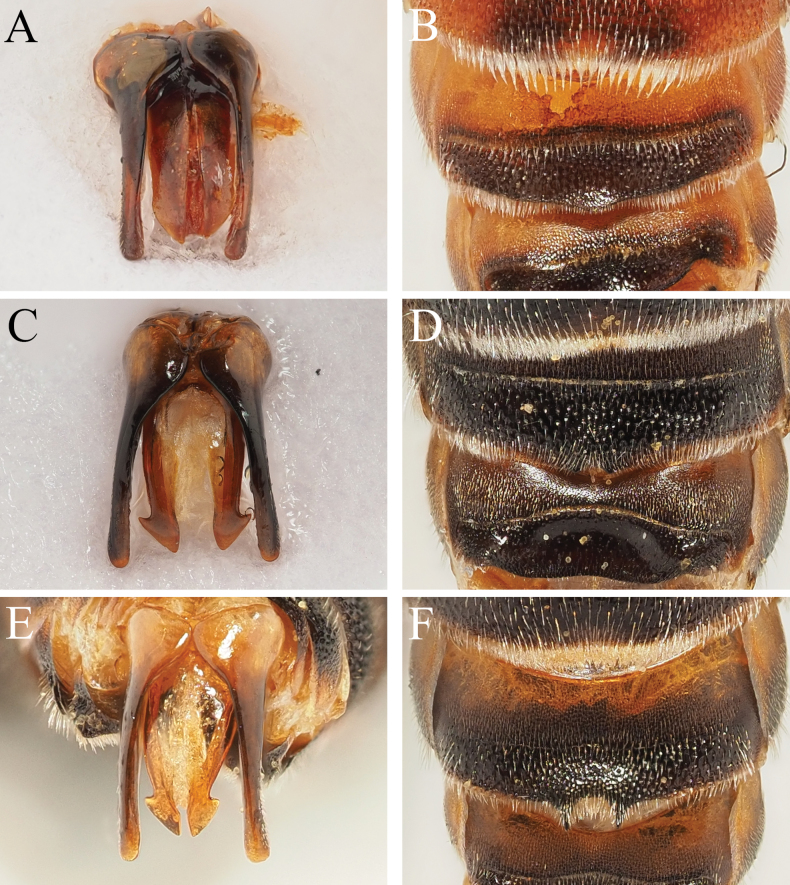
Male *Dioxys* species, genital capsule and S4 **A, B***Dioxysrufipes* Morawitz, 1875 (Greece) **C, D***Dioxyscinctus* (Jurine, 1807) (Spain) **E, F***Dioxysrotundatus* Pérez, 1884 (Morocco).

#### ﻿2. Dioxys atlanticus

Taxon classificationAnimaliaHymenopteraMegachilidae

Saunders, 1904

247EE566-F026-54FB-8AA5-389D9F0FA9A7

Figs 5A–F , 6A–F

Dioxys atlantica Saunders, 1904: 232, ♀♂ [Spain: Tenerife, NHMUK, examined].

##### Material examined.

**Figure 5.**
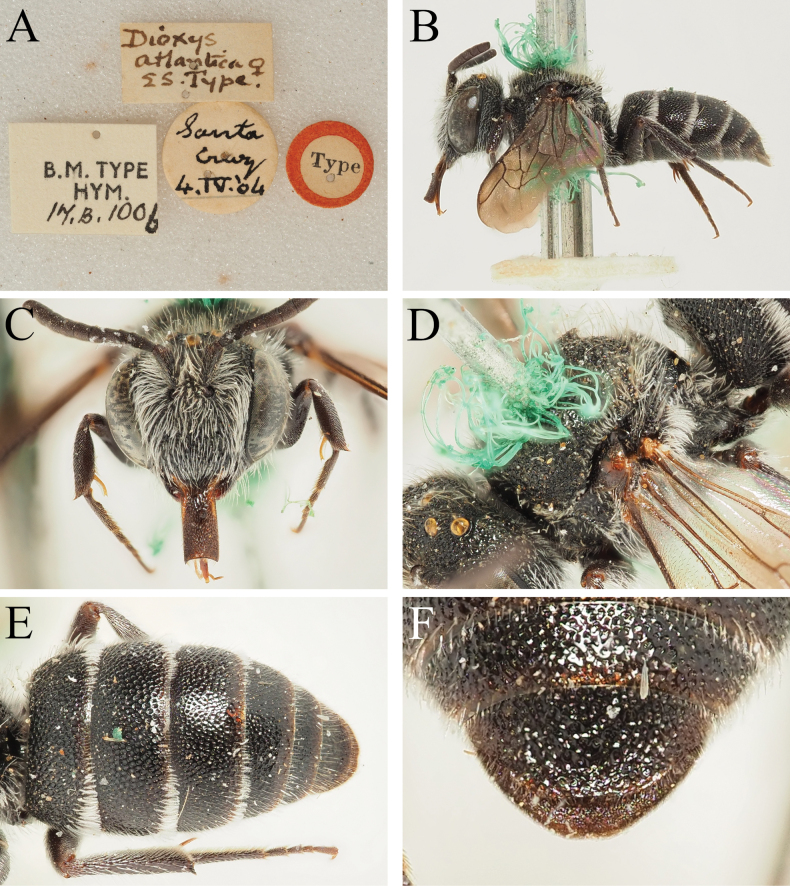
*Dioxysatlanticus* Saunders, 1904, female syntype (NHMUK) **A** label details **B** habitus, profile view **C** head, frontal view **D** mesosoma, dorsolateral view **E** terga, dorsal view **F** T6, dorsal view.

**Figure 6.**
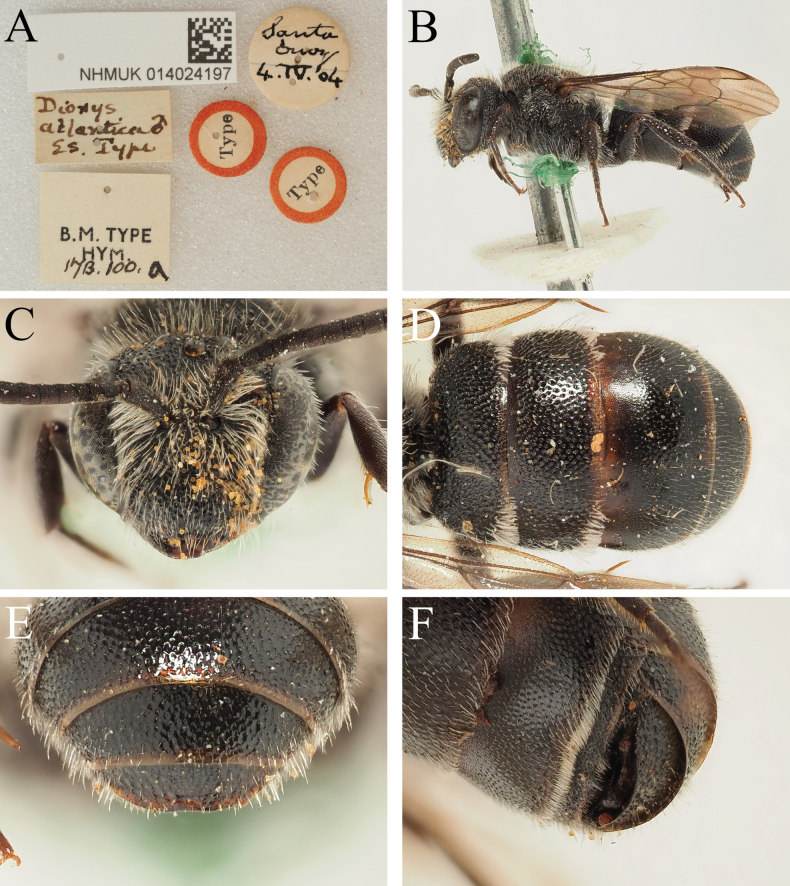
*Dioxysatlanticus* Saunders, 1904, male syntype (NHMUK) **A** label details **B** habitus, profile view **C** head, frontal view **D** terga, dorsal view **E** T4–T6, posterior view **F** T2–T4, ventrolateral view.

**Spain** • 1♂, 1♀; Santa Cruz [Tenerife]; 4 Apr. 1904; A.E. Eaton leg.; NHMUK (***syntypes***) (Figs 5A–F, 6A–F) • 1♀; Gran Canaria, Santa Lucia [Santa Lucía de Tirajana]; 800 m a.s.l.; 15 Jan. 2001; H. & I. v. Oorschot leg.; RMNH; ZMA.INS.5142848 • 1♀; Gran Canaria, Bco. Tasartea [Barranco de Tasarte]; 21 Mar. 1987; F. de la Roche leg.; B. Tkalců det.; OÖLM.

##### Remarks.

Discussion of the species and its distribution is required. In addition to Tenerife and Gran Canaria, Bogusch (2023) mentioned also Lanzarote citing Hohmann et al. (1993), Egypt citing Warncke (1977), and newly recorded the species from Sardinia based on novel records. Each of these points requires dissection.

The record(s) from Lanzarote are difficult to interpret because of the subsequent description of *D.lanzarotensis* (see Section 8). It is possible that specimens of the then unknown *D.lanzarotensis* were unwittingly determined as *D.atlanticus*, since the two are similar in size and colouration; the type specimen of *D.lanzarotensis* was captured on 3 March 1987 (Tkalců 2001: 49), it may have formed the basis of the report of Hohmann et al. (1993). The records from southern Egypt (Luxor, Abydos Baliana), as written, do not make ecological or morphological sense. Warncke (1977: 278) says that the morphological differences between *D.atlanticus* and North African *D.cinctus* (Jurine, 1807) are extremely slight, citing slightly finer punctation of T3–T6, that the female S6 is more rounded, and the male has similarly long pointed teeth medially on the margin of S4. Examination of the two syntypes of *D.atlanticus* raise the question as to whether Warncke ever examined them, because the shape of T6 in *D.atlanticus* is clearly substantially more rounded than in *D.cinctus* (compare Fig. 5F with Fig. 1A), as is the margin of S6, whereas North African (and indeed, all) *D.cinctus* have S6 rectangular in outline. The male of *D.atlanticus* does indeed have long pointed teeth medially on S4 (Fig. 6F), but this places it much closer to *D.rotundatus* Pérez, 1884 (Fig. 4F; this name is re-established as the senior synonym of *D.moestus* Costa, 1884, see Section 11), as *D.cinctus* males only have slight bumps on the margin of S4 (Fig. 4D). I concur with Bogusch (2023) in rejecting the combination of D.cinctusssp.atlanticus proposed by Warncke (1977). The morphology of these specimens is discussed below.

Finally, the new records of *D.atlanticus* from Sardinia are doubly questionable. The first is because there is not a single insect species which has a distribution of the Canary Islands and Sardinia, without records from North Africa, and more pertinently because of the original description of *D.moestus* which has a *locus typicus* of Sardinia (Costa 1884). The description is short, in Latin and Italian, and it is worth reproducing in full here:

“*Dioxysmoesta*. – D. nigra unicolor, cinerea pubescens, abdominis segmentis primis quatuor vel quinque postice fasciola e pilis stratis albis cinctis. – Long. mill. 4.”

Molto diversa dalla pyrenaica non solo per avere egualmente neri tutti gli anelli addominali, ma anche per le dimensioni minori ed il sesto anello addominale più semicircolare”.

[*Dioxys* entirely black, pubescence grey, the first four to five abdominal segments posteriorly covered with layers of white hair.

Very different from pyrenaica not only for having all of the abdominal segments entirely black, but also for its smaller size and the sixth abdominal segment more semi-circular.].

Costa clearly describes an all-black species that is smaller than *D.pyrenaicus* Lepeletier, 1841 [= *D.cinctus*] and has the final segment of the abdomen [= T6] more semi-circular. This description can only correspond to *D.rotundatus*, and corresponds to the black specimens of “*D.atlanticus*” found on Sardinia. This strongly suggests that the all-black Sardinian specimens simply are *D.rotundatus* as described by Costa (1884) as *D.moestus*. Moreover, specimens from Sardinia are not always entirely black. Warncke (1977: 275) writes that:

“♀ San Lussurgio/Sardinien, Lectotypus Mus. Napoli. Die Beschreibung kennzeichnet eine vollkommen schwarz gefürbte kleine *Dioxys*-Art. Das stimmt nicht, das 2. Tergit ist vollständig rot und die Seiten des 1. und 3. Tergits sind trübe rot!, womit das Tier auch in den übrigen Merkmalen mit *Dioxysrotundata* übereinstimmt!”

[The description indicates a small and completely black *Dioxys* species. This is not true, as the second tergum is entirely red and the sides of the first and third terga are dull red!, which means that the animal agrees with *Dioxysrotundata* in other characteristics].

This raises the question as to whether or not Warncke’s “lectotype” is actually valid since it does not match the description (cf. Schwarz et al. 1996), but this is of secondary importance and the overall comment combined with examination of new specimens from Sardinia (kindly loaned by Petr Bogusch, see material examined in Section 11. *Dioxysrotundatus*) indicates considerable colour variation. One female from Sardinia has T2 dull red, almost black, and a second female has T2 entirely black. The question is therefore, is *D.atlanticus* morphologically distinct from a widespread *D.rotundatus* which can be entirely black or have a metasoma marked with red, with variation between these two states?

I agree with Bogusch (2023) that *D.atlanticus* and *D.rotundatus* (referred to as *D.moestus*) are very morphologically similar; this is most apparent in the males which both possess S4 with a pair of long projecting teeth medially (Figs 4F, 6F), together these emphasising the median emargination. Based on the small number of specimens that I have been able to examine, the only clear structural character that I can see to allow separation of these species is the sculpture of the apical tergal segments, as well as the width:length ratio of the disc of T6. In female *D.atlanticus*, the punctures of T6 are very shallow and placed closely together, making it difficult to distinguish between punctures and shiny interspaces (Fig. 5F); in the male, the punctures of T5 and T6 almost disappear due to their shallowness (Fig. 6E). In contrast, in female *D.rotundatus* the apical tergal segments have the punctures deeper and slightly more spaced, therefore clearly and unambiguously contrasting the shiny interspaces (Fig. 1B); in the male, the punctures of T6 remain clear and distinct.

**Figure 7.**
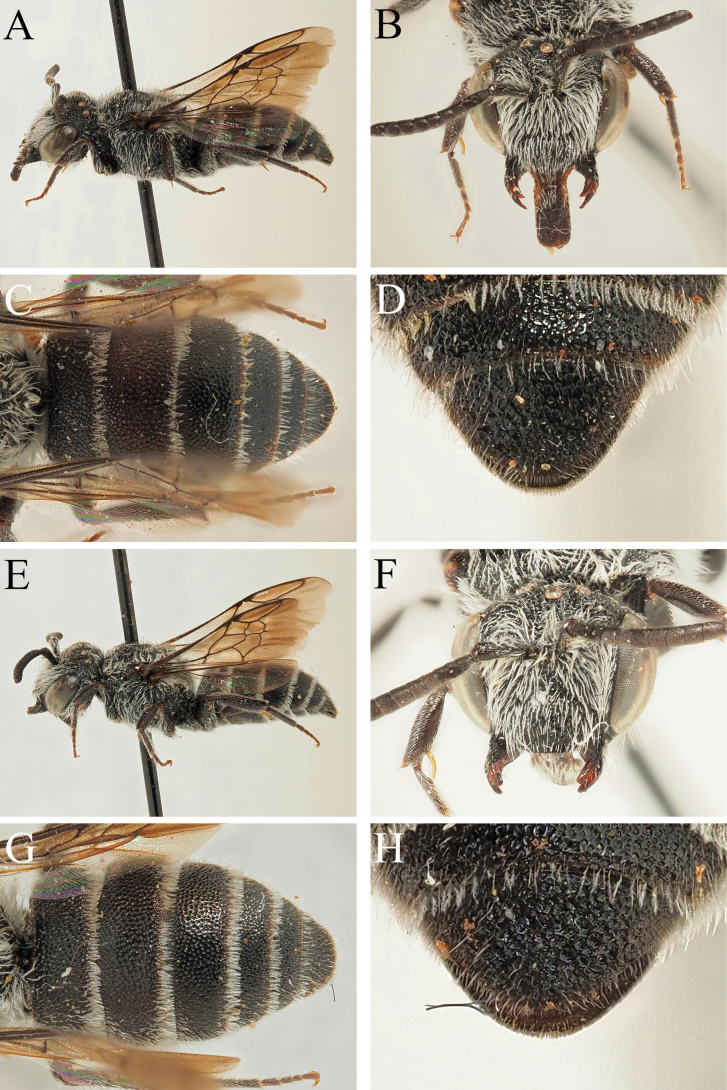
*Dioxysrotundatus* Pérez, 1884 from Sardinia **A–D** female 1 **E–H** female 2 **A** habitus, profile view **B** head, frontal view **C** terga, dorsal view **D** T6, dorsal view **E** habitus, profile view **F** head, frontal view **G** terga, dorsal view **H** T6, dorsal view.

The two Egyptian specimens examined by Warncke were also sent to me on loan (see material examined in Section 11. *Dioxysrotundatus*), representing a female and a male. The female has T2 entirely red-marked, and T6 is rounded with the punctures clear and well-defined, with shiny interspaces. In this regard it clearly matches *D.rotundatus*. The male has S4 with relatively short teeth flanking the medial emargination, making it somewhat intermediate between the condition found in *D.cinctus* and *D.rotundatus/atlanticus*, but the punctation of T5 and T6 is strong and well-defined, not disappearing into the integument. Measurement of the width:length ratio of the disc of the female T6 (from maximum visible width and maximum visible length of the disc in dorsal view) produces values of 1.82–1.90:1 for *D.rotundatus* (Figs 1B, 7D, H) and 2.12:1 for the syntype of *D.atlanticus* (Fig. 5F). Even though the punctation of T6 of one of the Sardinian specimens is more chaotic and begins to resemble that of *D.atlanticus* (Fig. 7H), the overall width:length ratio of T6 is 1.90, and less than that observed in *D.atlanticus*. This means that neither the female Sardinian specimens or the female or male Egyptian specimens are referable to *D.atlanticus* in the narrow sense used here.

With access to suitable material, these characters (T6 punctation and width:length ratio) can be used consistently. Therefore, I take the position that *D.atlanticus* is restricted to Tenerife and Gran Canaria, as I have not seen any specimens with equally weak punctation on the apical tergal segments elsewhere in the western Mediterranean or North Africa, the distributional range of *D.rotundatus* (see Section 11).

**Figure 8.**
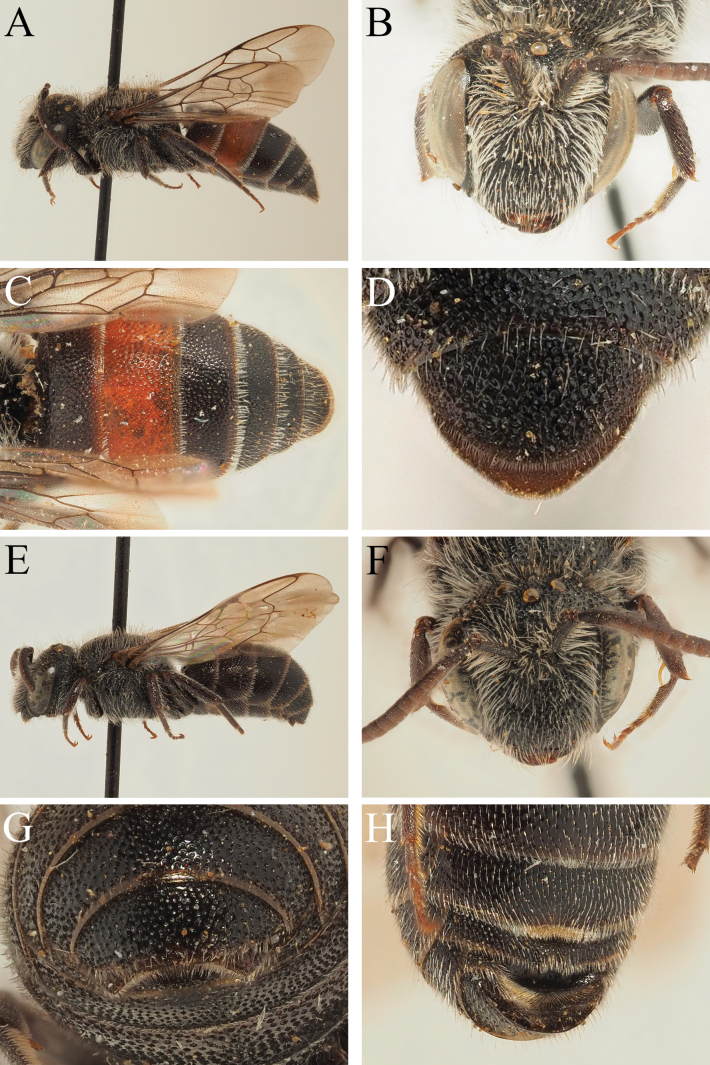
*Dioxysrotundatus* Pérez, 1884 from Egypt **A–D** female from Luxor **E–H** male from Abydos **A** habitus, profile view **B** head, frontal view **C** terga, dorsal view **D** T6, dorsal view **E** habitus, profile view **F** head, frontal view **G** T5 and T6, posterior view **H** S2–S4, ventral view.

##### Distribution.

Spain (Tenerife, Gran Canaria) (Saunders 1904).

#### ﻿3. Dioxys chalicodus

Taxon classificationAnimaliaHymenopteraMegachilidae

Lucas, 1849

6996E78E-0208-553A-92A2-7D13E863732A

Dioxys chalicoda Lucas, 1849: 207, ♀♂ [Algeria, MNHN, not examined].

##### Material examined.

**Egypt** • 1♀; Matariele [El Matareya]; 7 Mar. 1915; A. Alfieri leg.; J.D. Alfken det.; USNM; **Israel** • 1♂; Arava, 4 km W of Hazeva; 10 Mar. 1990; R. Leys leg.; RMNH; RMNH.INS.1660492; **Libya** • 1♂; Bengasi; 12 Feb. 1931; MSVI; **Morocco** • 1♀; Oriental, Guercif, Debdou, 2 km S of Debdou; 1500 m a.s.l.; 11 May 2022; T.J. Wood leg.; TJWC; **Tunisia** • 1♂; Zarzis; 1–14 Feb. 1995; M. Boness leg.; OÖLM.

##### Distribution.

Morocco, Algeria, Tunisia, Libya, Egypt, Israel* (Lucas 1849; Popov 1936; Warncke 1977; Kuhlmann et al. 2014; Bogusch 2023; Wood 2023).

##### Distributional notes.

The record of the species from Egypt lacks precise specimen details (Kuhlmann et al. 2014), but can be confirmed through the USNM specimen. The species is newly recorded from Israel. Warncke (1977) reported the species from Gibraltar, but Bogusch (2023) notes that this was a misidentification of *D.ardens*, with the specimen currently in collection MSCA, to be deposited in the OÖLM collection (P. Bogusch, pers. comm. December 2024).

#### ﻿4. Dioxys cinctus

Taxon classificationAnimaliaHymenopteraMegachilidae

(Jurine, 1807)

215C1CC2-2978-5832-9796-BA237A5B920F

Trachusa cincta Jurine, 1807: 253 [no type material].
Dioxys pyrenaica Lepeletier, 1841: 515, ♀♂ [France, MNHN, not examined]..
Dioxys maura Lepeletier, 1841: 516, ♀ [Algeria, ?MNHN, not examined]
Dioxys cruenta Gerstäcker, 1869: 166, ♂ [Italy: Sicily, ZMHB, not examined].
Dioxys spinigera Pérez, 1884: 299, ♀♂ [France, MNHN, not examined].
Dioxys cincta var. jucunda Mocsáry, 1894: 36, ♀ [Hungary, HNHM, not examined].
Dioxys cincta ab. friederikae Mader, 1933: 125, ♀ [Austria, ?NMW, not examined].

##### Material examined.

**Algeria** • 7♀; W. Saida [Wilaya de Saida], Sidi Amar; 12 Apr. 1981; R. Leys leg.; ZMA.INS.5104007–ZMA.INS.5104013 • 5♂, 3♀; Alger; 5–19 Apr. 1898; F.D. Morice leg.; OUMNH • 1♀; Oran; 14 Apr. 1910; F.D. Morice leg.; OUMNH; **Bulgaria** • 1♂; Debar, Pervomaj; 14 Jun. 2017; B. Halada leg.; OÖLM • 1♀; S of Sozopol; 40 m a.s.l.; 11 Jun. 2017; M. Halada leg.; OÖLM; **Czechia** • 1♂, 1♀; Moravia, Bratčice, 20 km S Brno; 28 Jun. 2011; M. & Z. Halada leg.; OÖLM; **France** • 1♂; Auron/Alp. Marit.; 1700 m a.s.l.; 11 Jul. 1972; H. Wolf leg.; RMNH; RMNH.INS.1660520 • 1♂; Narbonne; 14 Apr. 1903; F.D. Morice leg.; OUMNH; **Greece** • 1♂; Central Greece, Galaxidi, 3.5 km NNW; 100 m a.s.l.; 7 Apr. 2024; T.J. Wood leg.; TJWC • 1♂; Central Macedonia, Stavros, 2 km N of Stavros; 22 May 2023; T.J. Wood leg.; TJWC • 1♀; Eastern Macedonia and Thrace, Kavala, 1 km N Ag. Andreas; 23 May 2023; T.J. Wood leg.; TJWC • 1♂; Kriti [Crete], Lasithi, Kato Zakros; 21 Apr. 1985; R. Leys leg.; ZMA.INS.5104021 • 1♂; Kriti, Rethimno, 2 km N Livadia; 27 Apr. 1985; R. Leys leg.; ZMA.INS.5104014 • 1♂; W. Creta, Phaistos; 26 Apr. 1983; H. Teunissen leg.; RMNH; RMNH.INS.1663101 • 1♂, 1♀; Centraal Gr., Chryson [Chryso]; 2 May 1984; G. & M. den Hollander leg.; RMNH; RMNH.INS.1663296–RMNH.INS.1663297 • 1♀; Corinth; 18 May 1912; F.D. Morice leg.; OUMNH • 4♂, 1♀; Olympia; 7–11 May 1901; F.D. Morice leg.; OUMNH • 4♂, 3♀; Zante [Zakynthos]; 16–19 May 1901; F.D. Morice leg.; OUMNH; **Hungary** • 1♀; 5 km N Veszprém; 4 Jul. 2016; B. Halada leg.; OÖLM • 1♂; Mor, Csákberény; 220 m a.s.l.; 24 May 2011; M. & Z. Halada leg.; OÖLM • 1♂; N of Várpalota; 11 Jun. 2020; M. Halada leg.; OÖLM • 2♀; Pakozd, E of Székesfehérvár; 4 Jul. 2018; M. Halada leg.; OÖLM • 1♂; Pakozd, E of Székesfehérvár; 12 Jun. 2020; M. Halada leg.; OÖLM; **Israel** • 1♂; Jerusalem; 11 May 1955; OÖLM; **Iraq** • 1♂; Dahuk, Bade; 1003 m a.s.l.; 15 May 2023; D. Baiocchi leg.; MSVI • 3♀; Kurdistan, Duhok gov., 5 km E of Ashewa, Mt. Gara; 1750–1950 m a.s.l.; 30 May – 12 Jun. 2024; D. Baiocchi leg.; MSVI; **Italy** • 2♂; Roma, Via Falcognana; 10 Jul. 1989; G.G.M. Schulten leg.; RMNH; ZMA.INS.5104015–ZMA.INS.5104016 • 2♂; Roma, Via Falcognana; 10 May. 1992; G.G.M. Schulten leg.; RMNH; ZMA.INS.5104017–ZMA.INS.5104018 • 1♂; Sicilia, Etna Z.W. wand; 27 Jun. – 2 Jul. 1976; J. Timmer leg.; RMNH; ZMA.INS.5104019; **Jordan** • 1♀; 20 km S of North Shuna, Tall Al Arbatin; 19 Apr. 1996; Ma. Halada leg.; OÖLM • 3♀; 25 km S At Tafila; 27 May 2007; Z. Kejval leg.; OÖLM • 1♀; Jordan Valley, Mubalath; 27 Apr. 1996; Mi. Halada; OÖLM • 4♀; N. Shuna; 20–30 Apr. 1996; Mi. Halada leg.; OÖLM • 1♂; Puglia, Lecce, Strada vecchia Frigole; 35 m a.s.l.; 25 May 2021; Bolino leg.; MSVI • 1♂, 1♀; Lazio, Maccarese; 30–65 m a.s.l.; 15 May 2021; D. Baiocchi leg.; MSVI • 1♂; Lazio, Viterbo, Norchia; 130–160 m a.s.l.; 12 May 2022; M. Selis leg.; MSVI; **Lebanon** • 1♀; Brumana [Broummana]; 30 Apr. 1899; F.D. Morice leg.; OUMNH; **Morocco** • 1♂, 2♀; Fès-Meknès, Taza, P5425, 3 km N of Galdamane; 780 m a.s.l.; 12 May 2022; T.J. Wood leg.; TJWC • 5♀; Fès-Meknès, Taza, R507, 2 km N of Ras El Ma; c. 750 m a.s.l.; 10 May 2022; T.J. Wood leg.; TJWC • 2♂; Oukaimeden, 50 km S Marrakech; 2700 m a.s.l.; 8 May 2015; Mucska leg.; OÖLM • 1♂; Souss-Massa, R105, Tizirt, 10 km N, Agadir N´ Guemzt env.; 12 Mar. 2022; T.J. Wood leg.; TJWC • 1♂; SW of Sefrou; 16 May 2003; M. Halada leg.; OÖLM; **Portugal** • 2♂, 1♀; Algarve, 200 m E of Cacela Velha; 200 m a.s.l.; 27 Apr. 2016; T.J. Wood leg.; TJWC • 1♂, 1♀; Algarve, Tavira, Forte do Rato; 22 Apr. 2016; T.J. Wood leg.; TJWC • 1♂; Algarve, Praia do Cabeço, near Monte Gordo; 26 Apr. 2016; T.J. Wood leg.; TJWC • 1♀; Guia; 27 Apr. 2008; D.W. Baldock leg.; TJWC; **Spain** • 1♂, 1♀; 10 km SE Baza; 9 May 2003; J. Halada leg.; OÖLM • 1♀; 20 km NE Ronda; 30 Apr. 2003; J. Halada leg.; OÖLM • 2♀; 50 km W Almería, Berja; 21–28 Apr. 2003; J. Halada leg.; OÖLM • 1♀; Embalase de Barbate Sw; 6–8 May 2017; Barták & Kubik leg.; OÖLM • 1♀; Granada, Sierra Nevada, El Dornajo; 1700 m a.s.l.; 6 Jun. 2021; T.J. Wood leg.; TJWC • 1♀; Granada, Sierra Nevada, Trevélez environs to Barranco Madrid; 1500–1700 m a.s.l.; 14 Jun. 2021; T.J. Wood leg.; TJWC • 1♂; La Joya, Almeria; 27 Mar. 1959; J. Suárez leg.; RMNH; RMNH.INS.1663103 • 1♂; Málaga, 5 km S Ronda; 29 May 1967; M.J. & J.P. Duffels leg.; RMNH; ZMA.INS.5104020 • 1♀; Málaga, Cortes de la Frontera, path to Llano de las Labores; 26 May 2021; T.J. Wood leg.; TJWC • 1♂, 1♀; Málaga, Gaucín, Gaucín to Puerto del Hacho; 1 Jun. 2021; T.J. Wood leg.; TJWC • 1♂, 1♀; Málaga, Júzcar, Júzcar to Sendero de la Eras; 29 May 2021; T.J. Wood leg.; TJWC • 1♂; Málaga, Pizarre [Pizarra]; 20 May 1967; M.J. & J.P. Duffels leg.; RMNH; ZMA.INS.5104022 • 1♀; Province Teruel, 20 km E Montalban; 1100 m a.s.l.; 19–20 Jun. 1991; J. Tiefenthaler leg.; OÖLM • 1♀; Sierra Filabres Albanchez; 23 Apr. 2003; J. Halada leg.; OÖLM • 1♀; Teruel, Albarracin; 1170 m a.s.l.; 12–19 Jun. 1994; A. Teunissen leg.; RMNH; ZMA.INS.5104005 • 2♂, 1♀; Algeciras; 1–30 Apr. 1905; F.D. Morice leg.; OUMNH • 1♂; Jimena [Jimena de la Frontera]; 1–30 Apr. 1905; F.D. Morice leg.; OUMNH • 1♂, 2♀; Vallvidrera; 1–31 May 1905; F.D. Morice leg.; OUMNH; **Switzerland** • 9♂, 1♀; Berisal; 28 Jun. – 2 Jul. 1895; F.D. Morice leg.; OUMNH; **Syria** • 1♀; 30 km S Suwayda, Dibbin; 15–17 May 1996; Ma. Halada leg.; OÖLM • 1♀; Salkhad env; 6 May 1996; Mi. Halada leg.; OÖLM; **Tunisia** • 1♀; 15 km W Mateur; 6 May 1984; J.P. Duffels leg.; RMNH; ZMA.INS.5104006 • 2♂; 30 km N Foum Tatahouine; 12 Feb. 1992; K. Warncke leg.; OÖLM • 1♂; Grombalia env.; 18 Mar. 1996; K. Deneš leg.; OÖLM • 1♂, 1♀; Makthar; 16–17 Apr. 1998; K. Deneš leg.; OÖLM • 1♀; Zaafrana; 6 Apr. 1999; K. Deneš leg.; OÖLM • 4♀; Carthage; 27 Apr. 1913; F.D. Morice leg.; OUMNH • 4♀; Carthage; 20 May 1910; F.D. Morice leg.; OUMNH • 3♂, 2♀; Hammam Bou Hadjar; 10 Apr. 1910; F.D. Morice leg.; OUMNH; **Turkey** • 1♂; Karadut env., 50 km NE Adiyaman; 1 Jun. 2001; K. Deneš leg.; OÖLM; **West Bank** • 1♀; Wadi el Kelt [Wadi Qelt]; 29 Mar. 1952; OÖLM • 1♂, 1♀; Bethlehem; 8 Apr. 1899; F.D. Morice leg.; OUMNH.

##### Distribution.

Found across central and southern Europe (including Bulgaria*), the Mediterranean basin (including Morocco, Algeria, Tunisia, and Libya), east to Israel, Lebanon*, Syria*, Jordan*, Turkey, Iraq*, the Caucasus, and the Pamir mountains in Central Asia (Popov 1936; Warncke 1977; Kuhlmann et al. 2014; Bogusch 2023). The species may be spreading northwards, with the first record from Germany made only in 2019 (Saure and Petrischak 2020). Separately, it is not impossible that Popov’s record from Central Asia actually corresponds to one of the three species he described (males are known only for *D.distinguendus*), as examination of a small number of male *Dioxys* specimens from Central Asia show a very similar genital morphology to *D.cinctus*. Further study of Central Asian species is required.

#### ﻿5. Dioxys cypriacus

Taxon classificationAnimaliaHymenopteraMegachilidae

Popov, 1944, sp. resurr.

B9448B69-A065-55D3-9AD5-FDB7DD583887

Dioxys cypriaca Popov, 1944: 121, ♀♂ [Cyprus, ZISP, not examined].

##### Material examined.

**Cyprus** • 3♂, 3♀; Limassol; 1–30 Apr. 1924; G.A. Mavromoustakis leg.; RMNH; RMNH.INS.1660491–RMNH.INS.1660496 • 1♀; Limassol; 29 May 1951; G.A. Mavromoustakis leg.; RMNH; RMNH.INS.1660497 • 1♂, 2♀; Limassol; 26 May 1956; G.A. Mavromoustakis leg.; RMNH; RMNH.INS.1660496–RMNH.INS.1660498 • 3♂, 2♀; Limassol; 21 Apr. 1957; G.A. Mavromoustakis leg.; T.J. Wood det.; RMNH; RMNH.INS.1660499–RMNH.INS.1660503.

##### Remarks.

Popov (1944) described *D.cypriacus* from Limassol (May 1935 and June 1936) based on specimens sent to him by George Mavromoustakis. Although I have not examined the type, specimens in the RMNH collection also come from Limassol and were labelled as “*D.cypriaca*” by Mavromoustakis, and so they are considered to be representative of the species. Popov compared *D.cypriacus* to *D.rufipes* based on the genital capsule, and due to the shape of the apexes of the penis valves which are produced into apical points in both species, but the outer margins of the penis valves are more parallel-sided in *D.rufipes*, and have the outer margin more strongly bulging in *D.cypriacus*. Moreover, *D.cypriacus* males have the scutal hairs short (typically as long as the length of a lateral ocellus), whereas in *D.rufipes* males the scutal hairs are long, clearly much longer than the diameter of a lateral ocellus.

Warncke (1977: 275) synonymised *D.cypriacus* with *D.pumilus*. However, he does not seem to have properly examined the male genital capsule of the taxa he placed either in synonymy or in combination with his broad species concept of *D.pumilus*. Across what can be called the *pumilus*-group, there is variation in the male genital capsule. Warncke (1977: 276, fig. 25) illustrates the genital capsule of *D.* “*pumilus*”, but this genital capsule does not match those displayed by topotypical specimens from the island of Rhodes (Fig. 3A). Four genital forms can be seen amongst members of this group. The most pertinent is that of the oldest name, *D.pumilus*; four males from Rhodos were dissected, along with another from the nearby island of Kos (see Section 10 for examined material). This capsule has the penis valves apically produced into triangular-like points, with a clear angle laterally on the outer margins (Fig. 3A). The genital capsule illustrated by Warncke is that of the form found in the western Mediterranean (*D.varipes* De Stefani) which has the penis valves apically broadened but without their apexes produced into triangular shapes (Fig. 3C). This matches the illustration of *D.maroccanus* Popov, 1936 (Popov 1936: 17, fig. 5) which is here placed in combination with *D.varipes* (see Section 13 for an explanation).

For *D.cypriacus*, the genital capsule also differs meaningfully from *D.pumilus*. Like *D.varipes*, the penis valves are apically broadened but without their apexes produced into triangular shapes; the lateral margin is simply bulging (Fig. 3E). Moreover, the apical margin of S4 in the male sex is emarginate in both *D.pumilus* and *D.cypriacus*. However, whilst in *D.pumilus* there is a clear tooth placed medially in the emargination, in *D.cypriacus* there is a very short and obscure tooth found in the emargination. The combination of differences in the genital capsule and sternal morphology allows *D.cypriacus* to be considered again as a distinct species.

##### Distribution.

Cyprus (Popov 1944).

#### ﻿6. Dioxys heinrichi

Taxon classificationAnimaliaHymenopteraMegachilidae

Warncke, 1977

FC846FD2-46B7-54AA-8361-9D24925EC4C9

Fig. 9A–F

Dioxys heinrichi Warncke, 1977: 275, ♀♂ [Algeria, MSCA, not examined].

##### Material examined.

**Figure 9.**
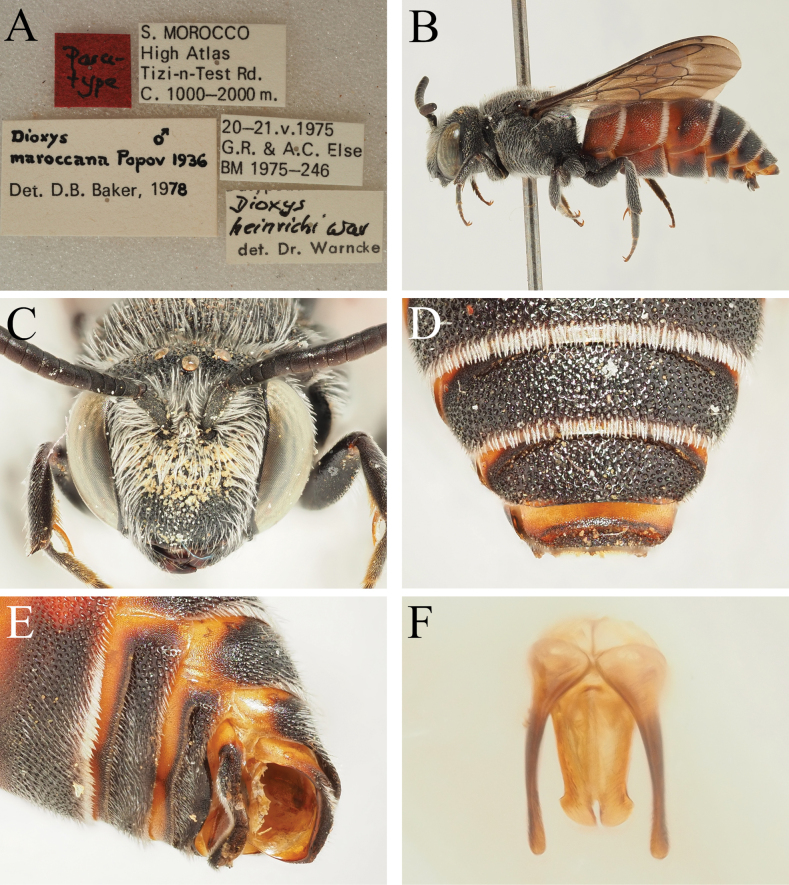
*Dioxysheinrichi* Warncke, 1977, male paratype (NHMUK) **A** Label details **B** habitus, profile view **C** head, frontal view **D** T4–T6, dorsal view **E** S3–S6, ventrolateral view **F** genital capsule, dorsal view.

**Morocco** • 1♂; S. Morocco, High Atlas, Tizi-n-Test road; 1000–2000 m a.s.l.; 20–21 May 1975; G.R. & A.C. Else leg.; K. Warncke det.; NHMUK (***paratype***; redetermined as *D.maroccana* by D. Baker, 1980) (Fig. 9A–F) • 1♂; T. Talrhemt [probably Jebel Talrhemt]; 28 May 1984; K.M. Guichard leg.; M. Schwarz det.; NHMUK.

##### Remarks.

*Dioxysheinrichi* was considered by Donald Baker to be conspecific with *D.maroccanus*, with this latter taxon therefore having priority. This position was never published, but Baker’s determination label is present on a paratype of *D.heinrichi* in the NHMUK collection (Fig. 9A). Examination of the holotype of *D.maroccanus* via photograph (Fig. 14A–D; see Section 13 on *D.varipes*) shows that Baker’s position was incorrect; *D.maroccanus* can clearly be separated from *D.heinrichi* due to the long scutal hairs of the latter species which are long and clearly exceed the diameter of a lateral ocellus in length (Fig. 9B), whereas they are clearly shorter than the diameter of a lateral ocellus in *D.maroccanus* (Fig. 14B). The main type series of *D.heinrichi* will be deposited in the OÖLM in the future.

##### Distribution.

Morocco and Algeria (Warncke 1977).

#### ﻿7. Dioxys hermonensis sp. nov.

Taxon classificationAnimaliaHymenopteraMegachilidae

979279C4-6FCD-5E29-8590-A729D8ED6940

https://zoobank.org/2B4C50B7-9101-4CD8-BB74-9C8C57E95015

##### Material examined.

***Holotype*: Israel** • 1♂; Mt. Hermon; 1500 m a.s.l.; 10 May 1975; K.M. Guichard leg.; NMHUK.

##### Diagnosis.

*Dioxyshermonensis* can be recognised as a *Dioxys* due to the metanotum with a conspicuous spine medially, the scutellum laterally produced into posteriorly projecting teeth, the axillae not produced into spines, body with pale to brownish hairs (not with long reddish hairs), first recurrent vein entering second submarginal cell, scutellum without visible carinae between lateral teeth and medial part of disc, labrum without transverse basal carina, and fore coxae with anterior surface rounded.

**Figure 10.**
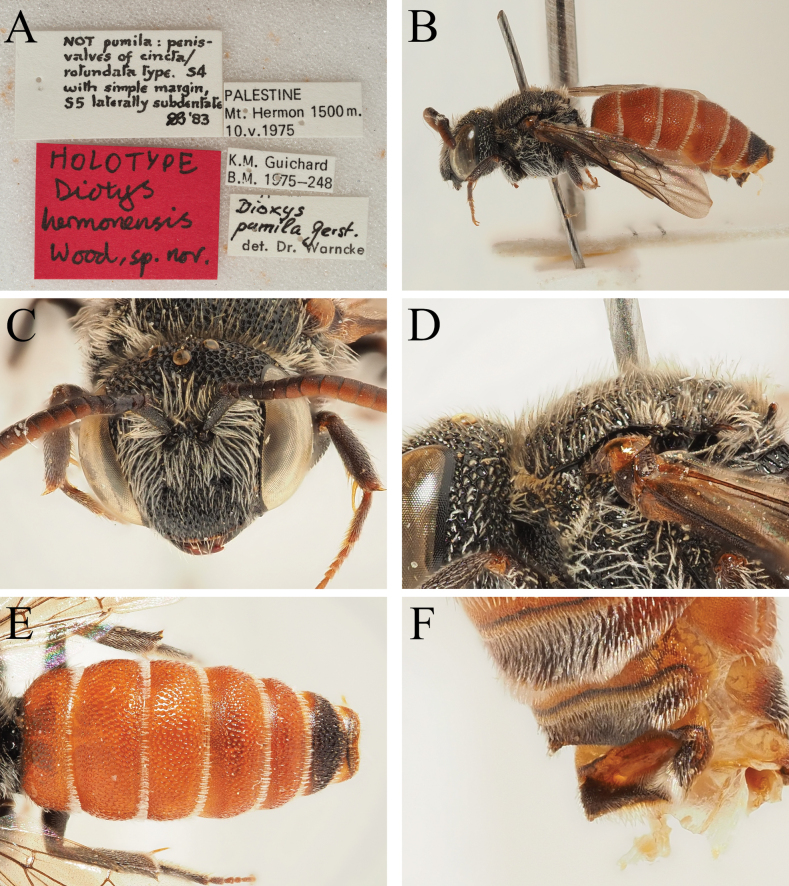
*Dioxyshermonensis* sp. nov., male holotype (NHMUK) **A** label details **B** habitus, profile view **C** head, frontal view **D** scutum, profile view **E** terga, dorsal view **F** S4–S6, ventrolateral view.

Within the *Dioxys*, *D.hermonensis* can be recognised due to the pale bodily pubescence and clear apical hairbands (Fig. 10E) combined with the scutum with short brownish hairs (Fig. 10B), these equalling or only slightly exceeding the diameter of a lateral ocellus. This places it in the *pumilus*-group of species (*cypriacus*, *pumilus*, and *varipes*). The genital capsule has the penis valves apically produced into triangular shapes, with a clear angle on the outer margin (Fig. 3G), whereas in *D.cypriacus* (Cyprus) and *D.varipes* (western Mediterranean) the apexes of the penis valves are thickened but without the outer margin showing a distinct angle (Fig. 3C, E). This places it closest to *D.pumilus* (Fig. 3A; eastern Mediterranean, including the Levant). *Dioxyshermonensis* can be easily separated due to the apical margin of S4 which is straight (Fig. 3H; in *D.pumilus* with the apical margin of S4 medially emarginate, this emargination displaying a small but distinct tooth medially, Fig. 3B) and due to the lateral margins of S5 which show a short and blunt but clearly distinct tooth (Fig. 10F; in *D.pumilus* with the lateral margins of S5 rounded, never showing an upstanding tooth). Currently, *D.hermonensis* is known only from a single specimen collected from the southern side of Mount Hermon.

##### Description.

**Female.** Unknown.

**Male.** Body length: 6.5 mm (Fig. 10B). ***Head***: Dark, 1.2 × wider than long (Fig. 10C). Clypeus strongly domed, densely punctate, punctures separated by 0.5 puncture diameters, interspaced raised and shiny. In frontal view, compound eyes with inner margins converging apically from point slightly above antennal insertions towards clypeus. Gena narrower than width of compound eye, in ventrolateral view with almost lamellate carina running from base of mandibles along ventral and posterior margin, becoming weak and obscure along posterior margin of vertex; ocelloccipital distance 1.5 × diameter of lateral ocellus. Face with moderately long and densely plumose white hair on paraocular areas and around antennal insertions, abruptly becoming sparse and obscure on clypeus and frons; longest hairs not equalling length of scape. Frons and vertex densely and deeply punctate, punctures separated by < 0.5 puncture diameters, interspaces shiny. Antennae basally dark, A6–A13 ventrally lightened orange; A3 slightly exceeding length of A4, clearly shorter than A4+5; A4 and A5 almost rectangular, almost twice as broad as long, remaining segments becoming progressively more elongate.

***Mesosoma***: Scutum and scutellum densely punctate, punctures confluent to separated by 0.5 puncture diameters, interspaces shiny. Scutellum laterally produced into short curved posteriorly projecting teeth, axillae laterally rounded, not produced into spines; metanotum medially with short spine. Lateral teeth of scutellum lacking visible carinae joining remaining disc of scutellum. Mesepisternum covered with large flat punctures, punctures separated by 0.5 puncture diameters, internal surface of punctures shiny; mesepisternum with moderately long, white, and densely plumose pubescence. Scutum and scutellum with short densely plumose brownish to whitish pubescence, length of hairs equalling or only slightly exceeding diameter of lateral ocellus (Fig. 10D). Fore coxae with anterior surface rounded. Legs dark, apical tarsal segments lightened reddish-brown, tarsal claws with small inner tooth. Wings hyaline to slightly brownish within cells; wings with two submarginal cells, second submarginal cell slightly larger than first submarginal cell; first recurrent vein enters second submarginal cell 3–4 vein widths from first transverse cubital vein.

***Metasoma***: Terga bright, T1–T5 entirely lightened orange-red, T6 with dorsal surface black (Fig. 10E). Sterna predominantly bright, S1 orange-red with black spot medially, S2 entirely orange-red, S3 predominantly orange-red with small black spot medially, S4–S5 predominantly dark with small orange-red areas laterally. Terga densely and regularly punctate, punctures separated by 0.5 puncture diameters. Terga with short apical hair fringes composed of pale plumose hairs, not obscuring underlying surface except for short distance laterally. S1–S4 with apical hair fringes, short on S1, S2, and S4, not exceeding diameter of lateral ocellus, long on S3, medially with hairs almost equalling 3 × diameter of lateral ocellus. Surface of S4 covered with plumose white hair, appearing felt-like; apical margin straight (Fig. 3H). S5 with lateral margins produced into short blunt teeth (Fig. 10F). Genital capsule with gonocoxae with inner margins forming obtuse angles, gonostyli long, parallel-sided, more or less featureless, outer margin finely hairy in apical 1/3 (Fig. 3G). Penis valves with outer margins apically converging, apexes produced into triangular shapes, with clear angle on outer margin.

##### Remarks.

This is the specimen reported by Warncke (1977) as *D.pumilus* from Mount Hermon – it bears a label reading “*Dioxyspumila* det. Dr. Warncke” (Fig. 10A). A label was added by Donald Baker in 1983 which reads: “NOT pumila: penis valves of cincta/rotundata type. S4 with simple margin, S5 laterally subdentate”. This astute observation allowed recognition of this species as distinct.

##### Etymology.

The name is taken from the name of Mount Hermon (Har Hermon), the *locus typicus*.

##### Distribution.

Israel (Mount Hermon). Likely present also on the Syrian and Lebanese parts of this mountain.

#### ﻿8. Dioxys lanzarotensis

Taxon classificationAnimaliaHymenopteraMegachilidae

Tkalců, 2001

456DE3B7-FE63-5C46-895C-DC8811F2BC00

Dioxys lanzarotensis Tkalců, 2001: 49, ♂ [Spain: Lanzarote, LRC, not examined].

##### Remarks.

Bogusch (2023) clarified the location of the type material, and provided important illustrations of the most important morphological features.

##### Distribution.

Spain (Lanzarote) (Tkalců 2001).

#### ﻿9. Dioxys montanus

Taxon classificationAnimaliaHymenopteraMegachilidae

Heinrich, 1977, sp. resurr.

78B48AB7-F79B-5016-9C02-D23F764CC71A

Fig. 11A–H

Dioxys montana Heinrich, 1977: 11, ♀♂ [Turkey, OÖLM, examined].

##### Material examined.

**Figure 11.**
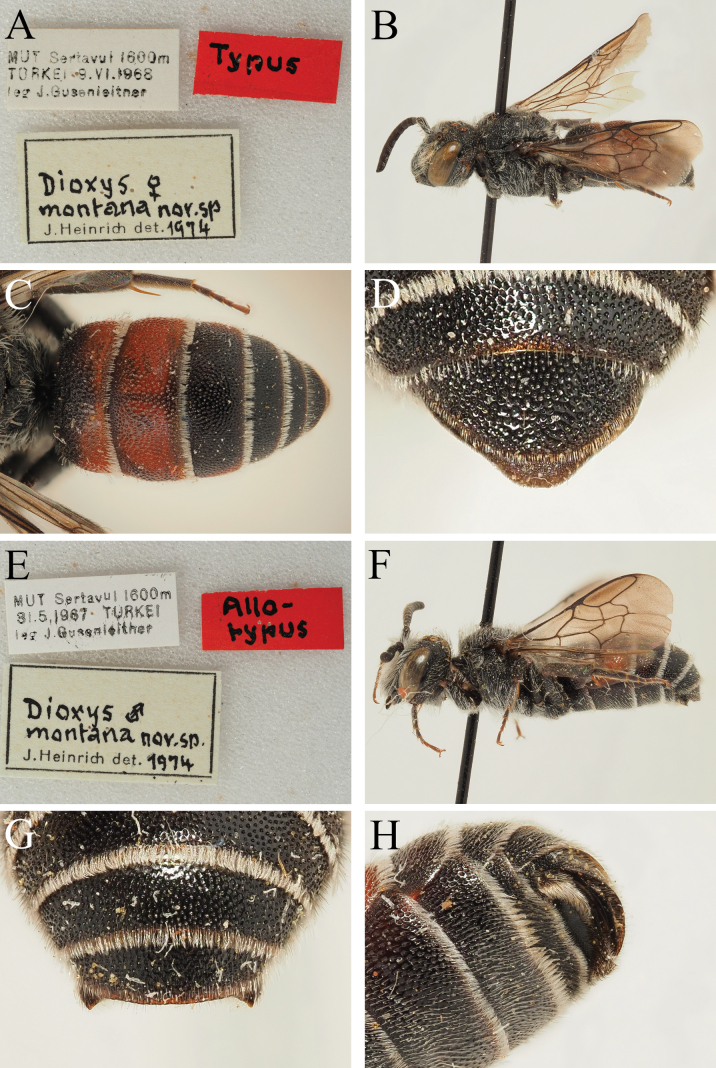
*Dioxysmontanus* Heinrich, 1977, female holotype (**A, B, C, D**) and male paratype (**E, F, G, H**) (OÖLM) **A** label details **B** habitus, profile view **C** terga, dorsal view **D** T6, dorsal view **E** label details **F** habitus, profile view **G** T4–T6, posterior view **H** S2–S4, ventrolateral view.

**Turkey** • 1♀; Mut, Sertavul [Sertavul Geçidi]; 1600 m a.s.l.; 9 Jun. 1968; J. Gusenleitner leg.; OÖLM (***holotype***) (Fig. 11A–D) • 1♂; Mut, Sertavul [Sertavul Geçidi]; 1600 m a.s.l.; 31 May 1967; J. Gusenleitner leg.; OÖLM (***paratype***) (Fig. 11E–H) • 1♀; Sille b. Konya; 8 Jun. 1972; K. Kusdas leg.; M. Schwarz det.; OÖLM • 1♂; Mut, Sertavul Pass [Sertavul Geçidi]; 22 Jun. 1976; J. Heinrich leg.; ZSM (***paratype***) • 1♂; Sille b. Konya; 9 Jun. 1975; J. Heinrich leg.; J. Heinrich det.; ZSM (labelled as paratype but not indicated as such in the original publication) • 1♂; Sille b. Konya; 4–17 Jul. 1977; J. Heinrich leg.; J. Heinrich det.; ZSM (labelled as paratype but not indicated as such in the original publication) • 1♂; Ürgüp As. Turk.; 26–28 Jun. 1977; J. Heinrich leg.; J. Heinrich det.; ZSM (labelled as paratype but not indicated as such in the original publication).

##### Remarks.

Bogusch (2023) synonymised *D.montanus* with *D.cinctus* with the justification that “Both type specimens … do not morphologically differ from typical specimens of *D.cinctus*”. No illustrations were provided. Based on examination of the type series, this position is unjustified, and *D.montanus* sp. resurr. is returned to species status. Specifically, the female T6 is semi-circular, with an evenly rounded outline, and S6 is more or less triangular, with the outer faces giving the impression of being slightly bowed or reflexed, before slightly curving outwards at their weakly truncate apex which projects beyond the apex of T6 in dorsal view (Fig. 11D). This is strongly different to the condition in *D.cinctus* where in dorsal view S6 can be seen as having a rectangular outline, clearly broader than long, and with the ventrolateral corners clearly visible on either side of T6 (Fig. 1A). Moreover, in the male, the apical margin of S4 is much more thickly hairy (Fig. 11H), with an apical hairband which is only slightly weaker than the hairband on S3 (in *D.cinctus* with the hairband of S4 clearly weaker and sparser than that on S3, Fig. 4D) and without a pair of short but projecting teeth, the apical margin broadly and widely emarginate (in *D.cinctus* with the apical margin of S4 straight, with a pair of short and projecting teeth, Fig. 4D).

##### Distribution.

South-western and central Turkey (provinces of Konya, Mersin, and Nevşehir; Heinrich 1977).

#### ﻿10. Dioxys pumilus

Taxon classificationAnimaliaHymenopteraMegachilidae

Gerstäcker, 1869

8E34286E-D956-5CF2-BAB6-E5C5CDEF259A

Dioxys pumila Gerstäcker, 1869: 167, ♂ [Greece: Rhodes, ZMHB, not examined].

##### Material examined.

**Greece** • 1♂, 5♀; Rhodes, Ixia s.l.; 6–18 May 1982; K.M. Guichard leg.; D. Baker det.; NHMUK • 1♂; Akrokorinth; 19 May 1964; W. Aigner leg.; OÖLM • 1♂, 1♀; Insel Kos, w.o. Kadamaina; 3–8 Jun. 2003; J. Tiefenthaler leg.; OÖLM • 1♀; Messinia, Kardamili [Kardamyli]; 24 May 1998; H. & J.E. Wiering leg.; ZMA.INS.5104023 • 1♂; Rhodos, Faliraki; 24 Apr. 1970; H. Teunissen leg.; RMNH; RMNH.INS.1663104 • 1♂; Rhodos, Kamiros [Kameiros]; 30 Apr. 1976; H. Teunissen leg.; RMNH; RMNH.INS.1663108 • 2♂; Rhodos, Rodini; 22 Apr. 1970; H. Teunissen leg.; RMNH; RMNH.INS.1663105–RMNH.INS.1663106 • 4♂; Rhodos, Moni Skadi bei Apolakkia; 22 Apr. 1998; P. Hartmann leg.; M. Schwarz det. 1999; ZSM • 2♀; Rhodos, west. V. Apolakkia, n. Monolithos; 23 Apr. 1998; M. Schwarz det. 1999; ZSM • 2♀; Kos; 10–14 May 2001; M. Blösch leg.; W. Arens det.; ZSM • 1♂; Olympia; 10 May 1901; F.D. Morice leg.; OUMNH; **Iran** • 1♂; Fars province, Dasht Arjan; 2040 m a.s.l.; 4 May 2016; M. Kafka leg.; OÖLM; **Jordan** • 2♀; Jordan Valley, Mubalath; 27 Apr. 1996; Mi. Halada leg.; T.J. Wood det.; OÖLM; **Syria** • 3♀; Salkhad env.; 6 May 1996; Mi. Halada leg.; T.J. Wood det.; OÖLM/TJWC • 1♀; 30 km S Suwayda, Dibbin; 15–17 May 1996; Ma. Halada leg.; OÖLM • 1♀; 40 km NE Damascus; 13 May 1996; Mi. Halada leg.; OÖLM • 2♀; Bloudan; 16 May 1995; K. Deneš leg.; OÖLM • 1♀; Ganawat [Qanawat]; 16 May 1995; K. Deneš leg.; OÖLM • 1♀; Maalula [Maaloula]; 17 May 1995; K. Deneš leg.; OÖLM; **Turkey** • 2♀; Findik [Fındık]; 1300 m a.s.l.; 28–29 Jun. 1991; K.M. Guichard leg.; M. Schwarz det.; NHMUK • 1♂; Ağrı, 20 km N Patnos; 1650 m a.s.l.; 29 May 1980; K. Warncke det & leg.; NHMUK • 3♂; Birecik As. Türk.; 17–19 May 1972; J. Heinrich leg.; OÖLM/ZSM • 10♀; Bolu, 17 km S Seben; 17 Jun. 1998; J. Halada leg.; OÖLM/TJWC • 1♂; Göreme; 23 Jun. 1993; K. Deneš leg.; OÖLM • 1♂; Gürün, 130 km S Sivas; 1600 m a.s.l.; 11 Jun. 2001; K. Deneš leg.; OÖLM • 1♀; Içel [Mersin], 12 km NW of Erdemli; 3–22 May 2001; I. Trojan leg.; OÖLM • 1♂; Mut; 30 May 1967; J. Gusenleitner leg.; OÖLM • 2♂, 2♀; Mut, As. Turk; 13–15 May 1972; J. Heinrich leg.; OÖLM/ZSM • 1♀; Siirt, 20 km NW of Sirnak [Şırnak]; 5 Jun. 1980; K. Warncke leg.; NHMUK • 1♂; Sille b. Konya; 8 Jun. 1972; J. Heinrich leg.; J. Heinrich det.; ZSM • 1♂; Gürün As. Türkei; 12–13 Jun. 1976; J. Heinrich leg.; J. Heinrich det.; ZSM • 1♂; Gürün As. Türkei; 30 May – 3 Jun. 1975; J. Heinrich leg.; J. Heinrich det.; ZSM • 1♂, 1♀; Ürgüp As. Turk.; 5–7 Jun. 1975; J. Heinrich leg.; J. Heinrich det.; ZSM.

##### Remarks.

The record from Israel (Mount Hermon) reported by Warncke (1977) is actually *D.hermonensis* sp. nov. (see Section 7). *Dioxyspumilus* is highly likely to be present in Israel given its presence in neighbouring Syria and Jordan, but this must be confirmed with verified specimens. Further to the comments made under *D.cypriacus* (Section 5), the concept of *D.pumilus* is used here much more narrowly than previous authors. The identification key and earlier comments above allow for the separation of male specimens that allow the geographic range to be clarified, as separation of females based only on morphological characters is challenging if not impossible, which is why a broad species concept for *D.pumilus* has been employed to date.

##### Distribution.

Greece, Turkey, ?Israel, Syria, Jordan*, Iran* (Gerstäcker, 1869; Heinrich, 1977; Warncke 1977 partim; Kuhlmann et al. 2014 partim; Bogusch 2023 partim).

#### ﻿11. Dioxys rotundatus

Taxon classificationAnimaliaHymenopteraMegachilidae

Pérez, 1884, sp. resurr.

BCF33A1C-6D24-5D3F-9AA1-A553C46B9533

Dioxys rotundata Pérez, 1884: 300, ♀ [Spain, MNHN, not examined].
Dioxys moesta Costa, 1884: 336, ♀ [Italy: Sardinia, IENU, not examined] syn. nov.

##### Material examined.

**Algeria** • 1♂, 2♀; Alger; 7–30 Apr. 1898; F.D. Morice leg.; OUMNH • 1♂, 1♀; Batna; 23 Jun. 1911; F.D. Morice leg.; OUMNH • 4♂, 15♀; Biskra; 10–23 Mar. 1920; K.J. & N.C.R. leg.; OUMNH; **Egypt** • 1♀; Louxor [Luxor]; 26 Feb. 1958; W.J. Pulawski leg.; MSCA • 1♂; Abydos, Rég. de Baliana [El Balyana]; 3–5 Mar. 1958; W.J. Pulawski leg.; MSCA; **Italy** • 2♀; Sardegna, Orroli; 2 Jun. 2011; G. Pagliano leg.; MSCA; **Morocco** • 1♀; Drâa-Tafilalet, Ouarzazate, P1506, Telouet, Adaha; 1700 m a.s.l.; 18 Apr. 2022; T.J. Wood leg.; TJWC • 2♀; Drâa-Tafilalet, Ouarzazate, P1507, 3 km SSE Irhels; 12 Apr. 2022; T.J. Wood leg.; TJWC • 2♀; Fès-Meknès, Azrou, 4 km SWW of Bakrit, Cascades Bakrit; 1650 m a.s.l.; 17 May 2022; T.J. Wood leg.; TJWC • 1♀; Fès-Meknès, Taza, P5425, 3 km N of Galdamane; 780 m a.s.l.; 12 May 2022; T.J. Wood leg.; TJWC • 1♀; Fès-Meknès, Taza, P5425, 3 km W of Aghil Oumial; 1300 m a.s.l.; 12 May 2022; T.J. Wood leg.; TJWC • 1♂; Meknes, Mt. Zerhoun; 13 May 1984; W. Perraudin leg.; OÖLM • 2♀; Oukaimeden; 2600–2800 m a.s.l.; 11 July 1975; J. Gusenleitner leg.; OÖLM • 1♀; S. Morocco, High Atlas, N. Tizi-n-Test; 2000 m a.s.l.; 24 Jun. 1974; G.R. Else leg.; NHMUK • 2♂, 1♀; Souss-Massa, R105, Tizirt, 10 km N, Agadir N´ Guemzt env.; 12 Mar. 2022; T.J. Wood leg.; TJWC • 1♂; Souss-Massa, R105, Tizirt, 8 km N, Ighir Ifran env.; 12 Mar. 2022; T.J. Wood leg.; TJWC • 1♀; Souss-Massa, Tafraoute, Azrou Ouado, 2 km W; 13 Mar. 2022; T.J. Wood leg.; TJWC • 1♂; Souss-Massa, Tizi N’Test; 16 Apr. 2024; D. Baiocchi leg.; MSVI • 1♀; Hoher Atlas, Oukaimeden; 2800 m a.s.l.; 11 Jul. 1975; A.W. Ebmer leg.; ZSM • 1♀; Nador; 14 Apr. 1990; M. Halada leg.; ZSM; **Spain** • 1♀; Cuenca, Huerta del Marquesado, environs north of town; 26 Jun. 2021; T.J. Wood leg.; TJWC • 1♀; 25 km SW Cartagena; 12 May 2003; J. Halada leg.; OÖLM • 1♀; 50 km W Almería, Berja; 21 Apr. 2003; J. Halada leg.; OÖLM • 1♂; Al. [Alicante], Benidorm; 26 Apr. 1997; V. Lefeber leg.; RMNH; RMNH.INS.1660524 • 1♀; Cigales, Valladolid; 30 Jun. 1982; E. Asensio leg.; RMNH; RMNH.INS.1660518 • 1♀; Granada, Sierra Nevada, Omg. Albergue Universitario; 2500–2600 m a.s.l.; 16 Jul. 1953; C.A.W. Jeekel leg.; RMNH; ZMA.INS.5104004 • 1♀; prov. Malaga, Vélez-Málaga, 7 km N; 17 May 1960; exc. R.M.N.H. leg.; RMNH; RMNH.INS.1663102 • 1♀; Salobreña, Granada; 26 May 1986; W. Perraudin leg.; OÖLM • 1♀; Sierra Alhamilla, Lucainena; 25 Apr. 2003; J. Halada leg.; OÖLM • 1♀; Sierra Filabres Albanchez; 23 Apr. 2003; J. Halada leg.; OÖLM • 1♂, 1♀; La Garriga; 1–31 May 1903; F.D. Morice leg.; OUMNH; **Tunisia** • 1♂; 30 km N Foum Tatahouine; 12 Feb. 1992; K. Warncke leg.; OÖLM • 1♀; Zarzis; 22 Mar. – 3 Apr. 1983; H. Wolf leg.; OÖLM • 1♀; Hammam Bou Hadjar; 21 Apr. 1910; F.D. Morice leg.; OUMNH • 2♂; Cherahill [Cherahil]; 25 Mar. 1910; F.D. Morice leg.; OUMNH.

##### Remarks.

The situation concerning this species has been confused, in multiple ways. Nomenclaturally, prior to the work of Warncke (1977), authors used the name *D.rotundatus* (e.g. Popov 1936). Warncke designated a lectotype of *D.moestus* in the IENU, a lectotype of *D.rotundatus* in the MNHN, and based on the years of publication which he considered to be 1883 for *D.moestus* and 1884 for *D.rotundatus*, synonymised the latter with the former. However, this is not the correct year of publication for *D.moestus*. Examining the scanned copy of the 15^th^ volume of the Bullettino della Società Entomologica Italiana available from the Biodiversity Heritage Library, although nominally published in 1883, this volume was split into trimesters. Trimester I (January–March) has no specified publication date other than 1883. Trimester II and III (April–September) were published together on 25 September 1883, and Trimester IV (October–December) was actually published on 15 April 1884. The work of Costa (pages 332–341) was contained in this fourth trimester. Therefore, the work should rightly be Costa (1884). This is confirmed by Poggi (2008: 162) who listed 15 April 1884 as the publication date for Volume 15 Trimester IV.

Furthermore, the work of Pérez on the parasitic bees of France was published by the journal the Actes de la société Linnéenne de Bordeaux. The 37^th^ volume of this journal, containing the work of Pérez (pages 205–378), was nominally published in 1883. However, following Baker (1996), the work of Pérez was actually first published in three parts. Specifically, pages 205–256 in November 1883, pages 257–320 in February 1884, and pages 321–378 in October 1884. The year 1883 has been used as the publication date by other authors, for example Lieftinck (1968) for *Thyreustruncatus* (Pérez, 1883). However, this depends on the page of publication, and since *T.truncatus* was published on page 312, it should correctly be *Thyreustruncatus* (Pérez, 1884). As *D.rotundatus* was published on page 300, it was therefore included in the second part that came out in February 1884. Consequently, *Dioxysrotundatus* was published two months before *D.moestus*, and must take priority, returning to a pre-Warncke (1977) nomenclatural situation.

The more serious confusion is biological. Prior to the work of Warncke (1977), *D.rotundatus* was considered a western Mediterranean species, but Warncke newly reported the species from Croatia, Greece, and Israel, and Heinrich (1977) reported the species from Turkey. However, Warncke did not seem to examine the genital capsule of this species, and indeed seems not to have placed much emphasis on genital morphology since his identification key never mentions them (although see illustrations in Warncke 1977: 276). This is strange because Popov (1936) provided excellent illustrations, and the genital capsule can be very helpful in allowing specimen determination in *Dioxys* (e.g. Fig. 3A, C, E, G). Donald Baker did extract specimen genitalia, and realised that *D.rufipes* Morawitz, 1875 was present in Turkey and in Greece (Crete) based on NHMUK specimens (see Section 12). However, this information was never published.

*Dioxysrufipes* can be instantly separated from *D.rotundatus* through inspection of the genital capsule (Fig. 4A), since the penis valves evenly taper to form sharp apical points without lateral triangular projections, whereas in *D.rotundatus* the penis valves show clear lateral triangular projections (Fig. 4E). Based on examined material, *D.rufipes* is widespread in the eastern Mediterranean, and I have been able to examine no specimens conforming to *D.rotundatus* from this region. Therefore, I consider *D.rotundatus* to be a western Mediterranean species extending east to Egypt in North Africa (see discussion in Section 2. *Dioxysatlanticus*) that is replaced by *D.rufipes* in the eastern Mediterranean.

##### Distribution.

Portugal, Spain, France, Italy (Sardinia), Morocco, Algeria, Tunisia, Egypt* (Warncke 1977 partim; Kuhlmann et al. 2014 partim; Baldock et al. 2018; Bogusch 2023 partim).

##### Distributional notes.

Records from Croatia, Greece, Turkey, and Israel (Heinrich 1977; Warncke 1977; Kuhlmann et al. 2014; Bogusch 2023) almost certainly refer to *D.rufipes* (see Section 12). Records from Croatia come from Kaštel Sućurac (Warncke 1977) which is in the south-east of the country and is much more likely to host eastern Mediterranean rather than western Mediterranean species, and so the judgement is made here that these are likely to refer to *D.rufipes*.

#### ﻿12. Dioxys rufipes

Taxon classificationAnimaliaHymenopteraMegachilidae

Morawitz, 1875

082DF7B4-4B88-51B5-BC63-1B2BB869D942

Dioxys rufipes Morawitz in Fedchenko (1875): 133 ♀ [Uzbekistan, ZISP, not examined].

##### Material examined.

**Greece** • 1♀; Crete, Ayia Galini s.l. [Agia Galini]; 3 May 1972; K.M. Guichard leg.; D. Baker det. 1983; NHMUK • 5♀; Crete, Paleochora s.l.; 10 May 1972; D. Baker det. 1983; NHMUK • 1♂; Kreta, N. Iraklion, Berg Giouchtas [Mount Juktas]; 22 Apr. 1990; E. Heiss leg.; OÖLM • 5♂; Prov. Florina, Amindeo [Amyntaio]; 700 m a.s.l.; 9–10 Jun. 1991; J. Tiefenthaler leg.; OÖLM • 1♂; Trikala; 17 Apr. 1962; K. Warncke leg.; T.J. Wood det. (det *D.rotundatus* by Warncke); OÖLM; **Jordan** • 1♀; Rawayshid [Ruwaished]; 23 Apr. 1996; Ma. Halada leg.; OÖLM; **Turkey** • 1♂; Mersin, Sertavul Gecidi; 4,500 ft a.s.l.; 22 Jun. 1960; Guichard & Harvey leg.; D. Baker det. 1983; NHMUK • 3♀; Urfa [Şanlıurfa]; 19–20 May 1967; J. Gusenleitner leg.; T.J. Wood det. (det *D.rotundatus* by J. Heinrich, 1974; det *D.varipes* ssp. by M. Schwarz, 1986); OÖLM • 1♂, 3♀; Urfa As. Türk. [Şanlıurfa]; 21–28 May 1972; J. Heinrich leg.; T.J. Wood det. (det *D.moestus* by M. Schwarz, 1985); OÖLM/ZSM • 1♀; Urfa As. Türk. [Şanlıurfa]; 22–26 May 1975; J. Heinrich leg.; T.J. Wood det.; ZSM • 1♂; Mut As. Turk; 13–15 May 1972; J. Heinrich leg.; T.J. Wood det.; ZSM• 1♀; As. Turk. [Elâzığ]; 9 Jun. 1976; J. Heinrich leg.; T.J. Wood det.; ZSM • 1♀; Birecik As. Türk.; 18 May 1967; J. Heinrich leg.; T.J. Wood det.; ZSM • 3♀; Uzuncaburç, 30 km N of Silifke; 28 May 1996; Mi. Halada leg.; OÖLM; **Uzbekistan** • 1♂; Kammashi; 18 Apr. 1931; Gussakovskij leg.; V. Popov det.; USNM • 1♀; Kammashi; 16 May 1931; Gussakovskij leg.; V. Popov det.; USNM.

##### Remarks.

**Figure 12.**
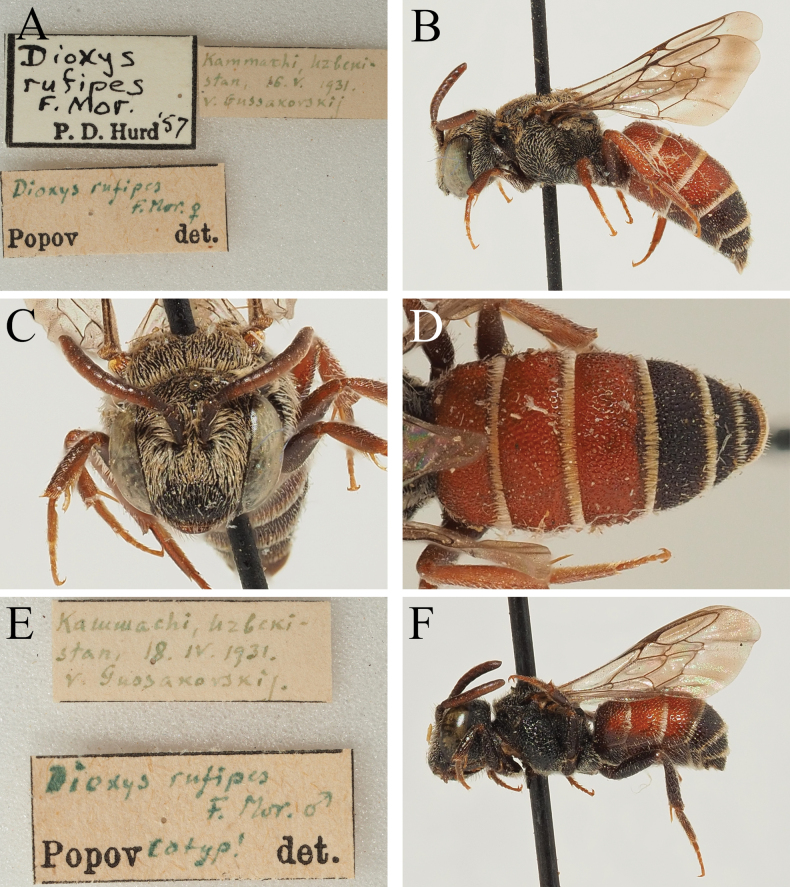
*Dioxysrufipes* Morawitz, 1875, female (**A, B, C, D**) and male (**E, F**) (USNM) **A** label details **B** habitus, profile view **C** face, frontal view **D** terga, dorsal view **E** label details **F** habitus, profile view.

*Dioxysrufipes* was described based on a single female collected in central Uzbekistan between Kattakurgan and Ulus (Fedchenko 1875). Popov (1936) examined several specimens from Uzbekistan, and described the male, illustrating the distinctive genitalia. A male and female specimen determined by Popov were deposited in the USNM where they could be examined (Fig. 12A–F). Additionally, 24 specimens from Greece, Jordan, and Turkey could be examined which conformed to *D.rufipes*. This included some specimens from Turkey determined as *D.moestus* Heinrich (who reported the species from Turkey; Heinrich 1977), and a specimen from Greece (Trikala) that was original identified as *D.rotundatus* by Warncke and then reported as *D.moestus* in Warncke (1977). In line with the above comments for *D.rotundatus*, *D.rufipes* is considered to be an eastern Mediterranean species with a distribution that extends into Central Asia, and essentially corresponds to *D.rotundatus* sensu auctorum in this region.

##### Distribution.

?Croatia, Greece*, Turkey*, ?Israel, Jordan*, Uzbekistan (Popov 1936; Heinrich 1977 partim, as *D.moestus*; Warncke 1977 partim, as *D.moestus*; Bogusch 2023 partim, as *D.moestus*).

##### Distributional notes.

Following on from the comments made for *D.rotundatus*, records from Croatia and Israel (Warncke 1977; specimens not examined) cannot be confirmed, but are considered plausible based on the species concept of *D.moestus* used by Warncke, and the presence of *D.rufipes* in nearby countries. Additional specimen records from the SEMC from Crete (K.M. Guichard material determined by Baker) are available on GBIF.

#### ﻿13. Dioxys varipes

Taxon classificationAnimaliaHymenopteraMegachilidae

De Stefani, 1887, sp. resurr.

A11ED35A-1624-5A31-A31D-49AC2898442B

Figs 13A–F , 14A–D

Dioxys varipes De Stefani, 1887: 113, ♀♂ [Italy: Sicily, RMNH, neotype by present designation].
Dioxys maroccanus Popov, 1936: 16, ♂ [Morocco, ZISP, examined by photograph].
Dioxys falsificus Engel, 2023: 176, ♀♂ [Spain, SEMC, examined by photograph] syn. nov.

##### Material examined.

**Figure 13.**
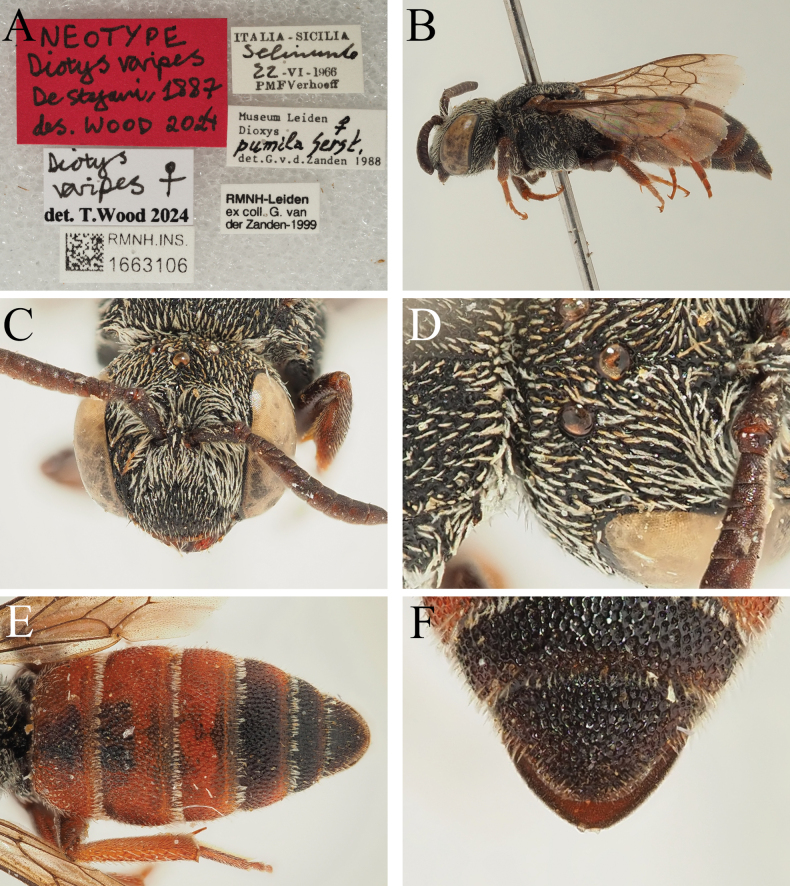
*Dioxysvaripes* De Stefani, 1887, neotype female (RMNH) **A** label details **B** habitus, profile view **C** face, frontal view **D** space between ocellar triangle and compound eye, frontal view **E** terga, dorsal view **F** T6, dorsal view.

**Figure 14.**
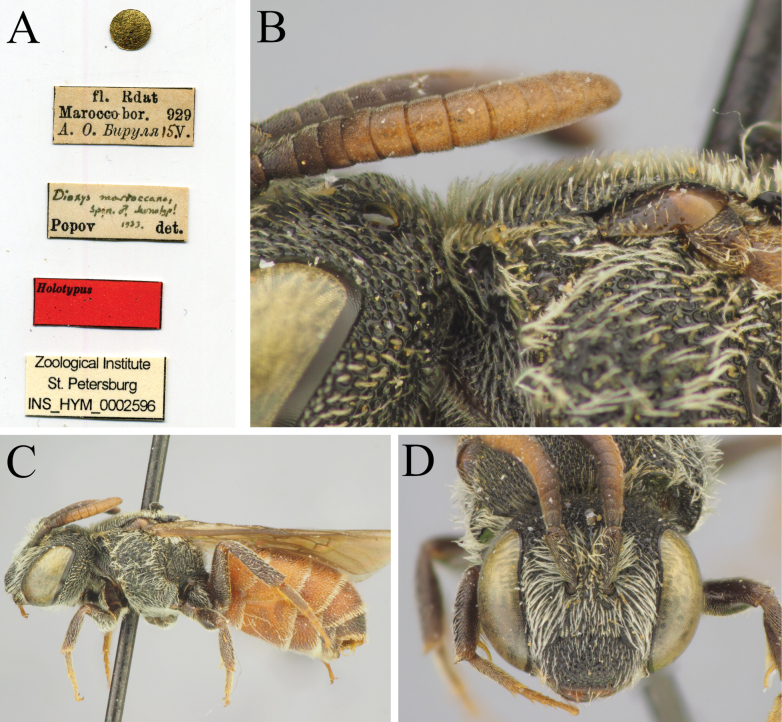
*Dioxysmaroccanus* Popov, 1936, female holotype (ZISP) **A** label details **B** scutum, profile view **C** habitus, profile view **D** face, frontal view.

**Italy** • ***Neotype***: 1♀; Sicilia, Selinunte; 22 Jun. 1966; P.M.F. Verhoeff leg.; RMNH; RMNH.INS.1663106 (Fig. 13A–F). **Italy** • 2♀; 35 km N Gela, NE Piazza Armerina; 27 May 2002; J. Halada leg.; OÖLM • 1♀; Sicilia, Caltagirone; 4–11 Jul. 1976; J. Timmer leg.; RMNH; ZMA.INS.5103998; **Libya** • 1♂; Cyrenaica, Brega [Mars el Brega]; 4 Mar. 1958; K.M. Guichard leg.; NMHUK • 1♀; Cyrene [Shahat]; 8 Jun. 1988; A. Četkovic leg.; RMNH; RMNH.INS.1663110; **Morocco** • 1♂; bor. [boreal = northern], River Rdat; 15 Mat 1929; A. Birula leg.; V. Popov det.; ZISP (***holotype*** of *Dioxysmaroccanus*; Fig. 14A–D) • 1♀; Fès-Meknès, Ahermoumou, P5407, immediately NW of Kassioua; 900 m a.s.l.; 15 May 2022; T.J. Wood leg.; TJWC; WPATW760-22 • 1♂; Fès-Meknès, Azrou, 4 km SWW of Bakrit, Cascades Bakrit; 1650 m a.s.l.; 17 May 2022; T.J. Wood leg.; TJWC • 1♂; Fès-Meknès, Boulemane, 5 km SE, junction of R503 and N4; 1900 m a.s.l.; 19 May 2022; T.J. Wood leg.; TJWC; WPATW761-22 • 1♂; Oudja, N-Jebel Fourhal, S-Ain Erreggada [Ain Reggada]; 500 m a.s.l.; 23 May 1994; M. Terzo leg.; RMNH; RMNH.INS.1660516 • 1♀; Souss-Massa, Tizi N’Test; 16 Apr. 2024; D. Baiocchi leg.; MSVI • 1♀; Azrou, (zedernwälder) [cedar forest]; 1660 m a.s.l.;17 Jul. 1975; A.W. Ebmer leg.; ZSM; **Portugal** • 1♂; Algarve, Forte do Rato, Tavira; 22 Apr. 2016; T.J. Wood leg.; TJWC • 2♂; Algarve, Montrigo; 9 Apr. 1988; J. Teunissen leg.; T.J. Wood det. (det. *D.varipes* by M. Schwarz, 1989); RMNH.INS.1660521; RMNH.INS.1660523 • 1♂; Algarve, Praia do Barril, near Tavira; 24 Apr. 2016; T.J. Wood leg.; TJWC • 1♂; Algarve, Praia do Cabeço, near Monte Gordo; 26 Apr. 2016; T.J. Wood leg.; TJWC; **Spain** • 1♀; Granada, Sierra Nevada, El Dornajo; 1700 m a.s.l.; 29 Jun. 2021; T.J. Wood leg.; TJWC • 1♀; Madrid, Pozuelo del Rey, 2 km NW; 10 Jul. 2021; T.J. Wood leg.; TJWC • 1♀; Madrid, Rivas-Vaciamadrid, Canal de Manzanares to Camino de Uclés; 19 May 2021; T.J. Wood leg.; TJWC • 1♂; Málaga, Arriate; 30 May 1967; M.J. & J.P. Duffels leg.; RMNH; ZMA.INS.5103999 • 1♀; Málaga, Ronda, 10 km NE, A-367; 25 May 2021; T.J. Wood leg.; TJWC • 1♂; Sevilla, Aznalcazar, S of Pinares de Aznalcazar; 21 May 2021; T.J. Wood leg.; TJWC • 1♀; Toledo, Toledo; 10 Jul. 1969; P.M.F. Verhoeff leg.; T.J. Wood det. (det. D.pumilusssp.varipes by Warncke); RMNH; RMNH.INS.1660522 • 1♀; Ronda; 12 Jun. 1998; A. Kroupa leg.; ZSM • 1♀; Chipiona; 8 Jun. 1998; A. Kroupa leg.; ZSM; • 1♀; Madrid; 29 Jun. 1910; G. Mercet leg.; OUMNH; **Tunisia** • 1♀; Tabarka, Tunis; 20 Apr. 1975; A. Mochi leg.; RMNH; RMNH.INS.1663111.

##### Remarks.

Further to the comments made under *D.cypriacus* and *D.pumilus*, *D.varipes* can be considered as a distinct western species within the *pumilus*-group. As the type material of *D.varipes* is lost, designation of a neotype is desirable. In line with the conditions of article 75.3 (ICZN 1999), this neotype is needed to clarify the taxonomic status of western Mediterranean populations of *D.pumilus* sensu lato, and to act as the senior name for the western population which is newly considered to be specifically distinct. Specific characters allowing its recognition are specified in the identification key and in Section 10 on *D.pumilus*. The Hymenoptera part of the De Stefani collection is considered to be lost (e.g., Gusenleitner and Schwarz 2002; Cornalba et al. 2024), and hence no-one has been able to retrieve the original material of *D.varipes* (e.g., Warncke 1977). The selected specimen is from Sicily, the original terra typica (De Stefani 1887), and morphologically conforms not only to the original description, but also to subsequent use of this species concept. Specifically, De Stefani describes the female as: “Piccola, ugualmente punteggiata in tutto il corpo; la testa ed il corsaletto neri rivestiti di breve pelurie cenerina più marcata sul volto… L’addome è rosso ferrugineol al primo, secondo e terzo segmento, spesso il quarto segmento comunemente nero è mischiato irregolarmente a del color rosso…” [Small, evenly punctured over the entire body, the head and mesosoma black and covered with short ash-coloured hairs, these more pronounced on the head… Metasoma red on terga 1–3, the 4^th^ segment is usually black but can be irregularly mixed with red…]. The body length was also given as 5½–6½ mm. This can only correspond to one species on the island of Sicily, namely the western sister taxon that is morphologically very close to *D.pumilus*. The selected neotype matches this description in the distribution of ashy hairs (Fig. 13B–D) and the colouration of the terga (Fig. 13E, F). The neotype is deposited in the RMNH collection.

Finally, it is necessary to deal with the taxon *Dioxysfalsificus* Engel, 2023. Engel described this taxon based on two females and two males (all from Algeciras in the extreme south of southern Spain, collected by K.M. Guichard), comparing it to *D.pumilus*. However, the description of this taxon is unsupported, and is based on material separated by D. Baker as “undescribed” and which was deposited in the Snow Entomological Collection after his death. Understanding the taxon therefore requires both an understanding of West Palaearctic *Dioxys* and an understanding of the species concepts used by Baker, Warncke, and Popov.

Baker did not publish on *Dioxys* in a strict sense (though see Baker 1998 for work on Dioxyini), but he disagreed with Warncke’s creation of *D.heinrichi* from north-west Africa. Baker re-determined a male paratype of *D.heinrichi* (NHMUK) as *D.maroccanus* (Fig. 9A; see Section 6), indicating that he did not accept Warncke’s synonymy of *D.maroccanus* with D.pumilusssp.varipes. Examination of the holotype of *D.maroccanus* (Fig. 14A–D), the synonymy of Warncke is correct, as the type specimen shows very short scutal hairs (shorter than the diameter of a lateral ocellus), and the genital capsule is typical (gonostyli apically slightly broadened but without laterally projecting triangular teeth). Baker was therefore de facto operating under the position that there was no name available for western populations of “*D.pumilus*” in Morocco or Spain. Considering that the type material of *D.falsificus* comes from Algeciras and was collected by Guichard in 1974, that Warncke (1977: 275) lists Algeciras within the distribution of D.pumilusssp.varipes, and he was known to have revised Guichard’s material in the NHMUK, it is likely that Warncke inspected these specimens himself. They may have been taken by Baker from the NHMUK directly (temporary staff appointment 1981–1982; O’Toole 2006) before ending up in his personal collection which was then deposited in the SEMC.

Morphologically, the description of *D.falsificus* is based on very subtle morphological characters (female T1 without a latitudinal carina, female apex of T6 more broadly rounded, apical margin of S5 with minute medial emargination, male S6 with short longitudinal carina medially). Interpretation of the significance of these characters is difficult. Measurement of the width:length ratio of T6 produces values of between 1.5–1.6:1; specimens from the western Mediterranean have T6 slightly broader compared to *D.pumilus* and *D.cypriacus* (Fig. 1F, specimen illustrated is from Morocco, see identification key couplet 7), but this value is so similar that it is difficult to use. In any case, *D.falsificus* does not seem to differ from this broader western Mediterranean trend. Examined female specimens of *D.varipes* from Morocco and Italy (Sicily), *D.pumilus* from Greece (Peloponnese) and Syria, and *D.cypriacus* (Cyprus) show this subtle latitudinal carina on T1, which seems absent on the Iberian specimens that could be inspected. However, when present, the carina is not strongly produced and often disappears into the surrounding punctures. It is not clear if Engel inspected any other specimens from Iberia or Morocco to investigate how consistent these putative characters are. When considered against the difference observed in the genital capsule between *D.varipes* and *D.pumilus*, this weak carina on T1 is considered an inconsistent character with poor discriminatory power.

Moreover, Wood (2023) and Wood et al. (2024) presented COI barcode genetic data for “*D.pumilus*” (now considered under the name *D.varipes*) from central and southern Spain (Madrid, Granada), southern Portugal (Algarve), and northern Morocco (Fès-Meknès). Sequences form a group with some genetic divergence, with average separation of 3.08% (range 0.15–6.16%). Whilst variation within Iberian populations was low (average 0.20%), the highest divergence was actually seen between the two Moroccan specimens, at 6.16%. Iberian sequences were therefore separated from the two Moroccan sequences by 4.51% (range 4.41–4.56%) and 3.50% (range 3.34–3.65%). Consequently, due to this variation, the Moroccan sequences do not form a direct sister group to Iberian sequences, with the two Moroccan sequences instead adjacent so that three Iberian sequences plus one Moroccan sequence are sisters, plus one additional Moroccan sequence as sister to this grouping (see Wood 2023: fig. 5).

Care should be taken in interpreting these results. The two Moroccan specimens come from the same province, were separated by just 65 kilometres, and were caught four days apart (see examined material). However, the specimen from Ahermoumou (WPATW760-22) comes from a moderate elevation of 900 m a.s.l. in an area with Mediterranean climate and mixed wooded vegetation where olive is cultivated. In contrast, the specimen from Boulemane (WPATW761-22) comes from high elevation at 1900 m a.s.l. from dry steppe desert. The observed higher genetic diversity in North African populations (based on the two sequences individuals) does not correspond to an apparent morphological difference in Morocco specimens themselves or between Moroccan and Iberian specimens. In the absence of morphological differences, North African and Iberian specimens are considered to be conspecific.

In this context, the name *D.maroccanus* is available to be applied to Iberian populations, at least in principle. Even if the extreme western populations of the taxon in Morocco and Iberia were found not to be conspecific with *D.varipes* populations on Sicily, *D.maroccanus* could be applied to specimens in the first instance from northern Morocco, the *locus typicus.* Since Engel’s (2023) paper does not mention the work of Popov once, and has not published information concerning morphological variation in *D.pumilus* sensu lato more broadly, *D.falsificus* is considered to not have been convincingly separated from *D.maroccanus* (the position that *D.maroccanus* is not synonymous with *D.pumilus* sensu Warncke being a key assumption inherent in following the unpublished position of Baker). Moreover, since no additional material outside of the four type specimens was revised (or if it was, this was not mentioned), convincing evidence for *D.falsificus* being consistently morphologically separated from populations from Sicily is lacking. Therefore, *D.falsificus* is synonymised with *D.varipes* syn. nov.

##### Distribution.

Portugal, Spain, Italy (Sicily), Morocco, Algeria, Tunisia, Libya* (De Stefani, 1887; Popov 1936 as *D.maroccanus*; Warncke 1977 partim, as D.pumilusssp.varipes; Kuhlmann et al. 2014 partim, as *D.pumilus*; Baldock et al. 2018 as *D.pumilus*; Bogusch partim, as *D.pumilus*; Wood, 2023 as *D.pumilus*).

## ﻿Discussion

This contribution to the taxonomy of the genus *Dioxys* produced a total of 13 species present in the West Palaearctic, the greatest number of species currently recognised in this region. Numerous issues were detected, including incorrect publication dates, confusion surrounding the interpretation of type material, lack of consultation of original descriptions, and a lack of examination of the male genital capsule. All of these combined to produce blurry species concepts that led to confusion for subsequent workers, compounding the issue further. The revised species identification key presented here will hopefully remove this taxonomic impediment, and facilitate new work on this genus of bees.

The centre of diversity for Old World *Dioxys* is north-western Africa, with Morocco and Algeria each hosting six species (*D.ardens*, *D.chalicodus*, *D.cinctus*, *D.heinrichi*, *D.rotundatus*, and *D.varipes*), with a total of five species in Tunisia. Adding in the Canary Islands, two more species (*D.atlanticus* and *D.lanzarotensis*) can be added, meaning that 61.5% of the West Palaearctic fauna can be found in north-west Africa plus the Canary Islands. This region is also very rich in Megachilini and Osmiini (e.g. Lhomme et al. 2020; Müller 2022), and since these bees are the principal hosts of *Dioxys* species (Le Goff 2005; Westrich 2018; Bogusch et al. 2020; Saure and Petrischak 2020; Bogusch 2023; Wood 2023), this high *Dioxys* richness is logical. Given the high endemic species richness of Megachilidae in North Africa, it is likely that fresh studies will continue to uncover undescribed *Dioxys* (and Dioxyini) richness in this region.

## Supplementary Material

XML Treatment for Dioxys ardens

XML Treatment for Dioxys atlanticus

XML Treatment for Dioxys chalicodus

XML Treatment for Dioxys cinctus

XML Treatment for Dioxys cypriacus

XML Treatment for Dioxys heinrichi

XML Treatment for Dioxys hermonensis

XML Treatment for Dioxys lanzarotensis

XML Treatment for Dioxys montanus

XML Treatment for Dioxys pumilus

XML Treatment for Dioxys rotundatus

XML Treatment for Dioxys rufipes

XML Treatment for Dioxys varipes

## Footnotes

Note: morphological separation of the three following species is challenging in the female sex. Geographic context should be used, in association with concurrently active males which are easier to diagnose via examination of the genital capsule.

## ﻿Additional information

### Author ORCIDs

T. J. Wood https://orcid.org/0000-0001-5653-224X

### Data availability

All of the data that support the findings of this study are available in the main text.
